# Assessing the relative vulnerabilities of Mid-Atlantic freshwater wetlands to projected hydrologic changes

**DOI:** 10.1002/ecs2.2561

**Published:** 2019-02-08

**Authors:** Denice H. Wardrop, Anna T. Hamilton, Michael Q. Nassry, Jordan M. West, Aliana J. Britson

**Affiliations:** 1 Department of Geography, Pennsylvania State University, University Park, Pennsylvania 16802 USA; 2 Tetra Tech, Inc., Santa Fe, New Mexico 87505 USA; 3 EPA Office of Research and Development, Washington, D.C. 20460 USA; 4 Oregon Department of Environmental Quality Laboratory & Environmental Assessment Division, Hillsboro, Oregon 97124 USA

**Keywords:** climate change, Mid-Atlantic, vulnerability assessment, wetlands

## Abstract

Wetlands are known to provide a myriad of vital ecosystem functions and services, which may be under threat from a changing climate. However, these effects may not be homogenous across ecosystem functions, wetland types, ecoregions, or meso-scale watersheds, making broad application of the same management techniques inappropriate. Here, we present a relative wetland vulnerabilities framework, applicable across a range of spatial and temporal scales, to assist in identifying effective and robust management strategies in light of climate change. We deconstruct vulnerability into dimensions of exposure and sensitivity/adaptive capacity, and identify relevant measures of these as they pertain to the attributes of wetland extent and plant community composition. As a test of the framework, we populate it with data for three primary hydrogeomorphic wetland types (riverine, slope, and depression) in seven small watersheds across four ecoregions (Ridge and Valley, Piedmont, Unglaciated Plateau, and Glaciated Plateau) in the Susquehanna River watershed in Pennsylvania. We use data generated from the SRES A2 emissions experiment and MRI-CGCM2.3.2 climate model as input to the Penn State Integrated Hydrologic Model to simulate future exposure to altered hydrologic conditions in our seven watersheds, as expressed in two hydrologic metrics: % time groundwater levels occur in the upper 30 cm (rooting zone) during the growing season, and median difference between spring and summer mean water levels. We then examine the spatial and temporal scales at which each of the components of vulnerability (exposure and sensitivity/adaptive capacity) shows significant relative differences. Overall, we find that relative differences in exposure persist at a very fine spatial grain, exhibiting high variability even among individual watersheds in a given ecoregion. For temporal scale, we find strong seasonal but weak annual relative differences in exposure resulting from a magnification of summer dry-down combined with winter and spring wet periods becoming wetter. Sensitivities/adaptive capacities show significant differences among wetland types. A comparison between our anticipated hydrologic alterations under climate change and historical changes in hydrology due to anthropogenic disturbance indicates potential shifts in hydrologic patterns that are far beyond anything that wetland managers have experienced in the past.

## Introduction

Wetlands are a familiar and important feature of our environment, providing a portfolio of ecosystem services that are deemed critical for human health and well-being (Millennium Ecosystem Assessment 2005, Mitsch and Gosselink 2015). The name captures their defining and unifying characteristic—they are “wet,” described as transitional habitats between upland and aquatic habitats (Cowardin et al. 1979, Brinson 1993*a*, Burkett and Kusler 2000). Wetlands are aquatic ecosystems of national importance. They are one of the major natural ecosystems that provide services to people (e.g., see [de Groot et al. 2002, Zedler and Kercher 2005, Keddy et al. 2009], including biogeochemical cycling of CO_2_ [Bonneville et al. 2008, Langley et al. 2009], water storage and groundwater recharge [Acharya 2000, McCarthy 2006], and nutrient cycling [Zhao et al. 2004]). Managers seek to preserve these valued services through the protection and restoration of wetlands (Pitchford et al. 2012). From the perspective of the U.S. Environmental Protection Agency (EPA), wetlands are a key aquatic resource (among streams, rivers, and lakes) covered under Section 404 of the Clean Water Act (CWA), which attempts to ensure that wetland impacts are avoided or minimized, or, when unavoidable, compensated for. Impacts are not solely derived from activities directly inside the wetland boundary, such as dredging or filling; a number of studies have shown that anthropogenic activities in the surrounding landscape, such as agriculture, deforestation, and urban development, to name a few, have impacts on wetlands and the services they provide (Kentula et al. 2004, Brooks et al. 2006, Wardrop et al. 2007, Whigham et al. 2007), via changing hydrologic patterns, sedimentation, and nutrient enrichment (Wardrop and Brooks 1998, Mahaney et al. 2004, 2005).

Because hydrology (“wetness”) is a key parameter driving variations in wetland structural and functional characteristics across landscapes (Brinson 1993*a*), wetlands are considered particularly susceptible to climate change through the effects of changing temperature and precipitation patterns on surface and groundwater hydrology (Cox and Campbell 1997, Winter 2000, Poff et al. 2002, Dawson et al. 2003, Burkett et al. 2005, Erwin 2009, Pitchford et al. 2012, Garris et al. 2015, Mitsch et al. 2015). Increasing temperatures, altered patterns of precipitation, and related increases in evapotranspiration can result in changes in surface and groundwater levels, where a change in only a few centimeters can result in changes in wetland size, in loss of wetlands to drylands, or in conversions to other wetland types (Schouten et al. 1992, Burkett and Kusler 2000). Climate changes also interact with the aforementioned anthropogenic stressors such as land use change (Ghedini et al. 2015, Malanson et al. 2017), potentially exacerbating existing wetland problems.

Wetland classification that focuses on these aspects of hydrology has the potential to predict, or explain variation in, wetland response to climate change. For this reason, a variety of studies have utilized classification schemes based on hydrology and hydrologic context (e.g., landscape and/or geologic factors) as a basis for projecting responses to climate change, albeit in general terms (Gilvear and McInnes 1994, Winter 2000). A further refinement of hydrology-based classification is the hydrogeomorphic (HGM) classification system, which utilizes hydrology in combination with geomorphology (position in the landscape) and water source to categorize wetlands (Brinson 1993*b*), making HGM classification useful in defining functions (Smith et al. 1995) and associated ecosystem services (McLaughlin and Cohen 2013) among wetland types. Such a classification may prove useful in projecting patterns of susceptibility to climate change effects across a range of wetland types, potentially providing a meaningful context for assessment and comparison.

Despite a relatively broad consensus on the probability of some type of climate change impact on wetlands, characterizing the potential outcomes and risks from climate change and formulating management options to mitigate those risks are considered a significant scientific challenge (Weaver et al. 2013). This reflects the complexities that typically contribute to any climate change-related assessment, including high variability and uncertainty, issues of scale, and a diverse array of technical (e.g., climate modeling, hydrology, and ecological processes) as well as social, economic, and political inputs. The concept of vulnerability, defined here as the extent to which a species, habitat, or ecosystem is susceptible to harm from climate change impacts (Schneider et al. 2001), can incorporate such a spectrum of factors and uncertainties, and so has come to be valued as an approach for understanding the risks of climate change to natural systems (Adger et al. 2004, Fussel 2007, Polsky et al. 2007, Berkhout et al. 2014). If appropriately assessed, the concept of vulnerability can be used to compare relative risks among management targets (e.g., wetland types or units in this case) and develop adaptation practices to address those risks by either reducing impacts or increasing the ability to cope (Kelly and Adger 2000, Polsky et al. 2007, Julius et al. 2008; Stein et al. 2014, Wise et al. 2014).

A widely applied paradigm in current vulnerability literature is to deconstruct vulnerability into components of exposure, sensitivity, and adaptive capacity (Adger et al. 2004, Adger 2006, Polsky et al. 2007, Dawson et al. 2011, Glick et al. 2011, Watson et al. 2013, Beever et al. 2016). In the context of climate change, widely accepted definitions of these components are as follows: Exposure is the nature and degree to which a system is exposed to significant climate variations; sensitivity is the degree to which a system is affected, either adversely or beneficially, by climate-related stimuli; and adaptive capacity is the ability of a system to adjust to climate change (including climate variability and extremes), to moderate potential damages, to take advantage of opportunities, or to cope with the consequences (Glick et al. 2011).

Adaptive capacity includes the intrinsic abilities of a species or system to respond through phenotypic plasticity, genetic diversity, or applicable life history traits such as dispersal or colonization ability (Dawson et al. 2011, Beever et al. 2016). However, it also includes external factors that influence these intrinsic ones, such as structural barriers that limit dispersal, and overharvesting that limits genetic diversity (Glick et al. 2011, Beever et al. 2016). The separation of intrinsic and extrinsic factors of adaptive capacity can be useful when considering management options; intrinsic abilities can be considered as a “preexisting condition” (Glick et al. 2011) that adaptation measures such as restoration, removal of barriers, and management policies can affect. This aligns with discussions in the broader global change research community, where adaptive capacity can also include societal adaptation, which in this case is the ability to use human management to attenuate ecological impacts from an identified climate-related hazard (IPCC 2007, 2013; Bierbaum et al. 2014). It is this human adaptive capacity component that we aim to inform through the application of vulnerability assessment results, and thus, we characterize only the intrinsic, or biological, component of adaptive capacity in this study.

In practice, classifying and operationalizing exposure as a distinct component is relatively clear. However, the distinction between measures of sensitivity and adaptive capacity (biological portion only, as defined above) is less clear. Sensitivity is commonly thought of as degree to which the survival, persistence, fitness, performance, or regeneration of a species or population is dependent on the prevailing climate and is generally articulated as a physiological response. The biological portion of adaptive capacity can include the intrinsic abilities of a species or system to respond through phenotypic plasticity, genetic diversity, or applicable life history traits such as dispersal or colonization ability (Dawson et al. 2011, Beever et al. 2016). Yet all of these represent biological traits that are manifest in response to the hazard. It is the combination of these biological traits that dictate how the organism or system is going to respond, and many metrics of biological responses to a specified hazard can be interpreted as either or both components. So from an ecological perspective and for the purposes of this paper, we represent ecosystem response as the combination of sensitivity and adaptive capacity (represented as sensitivity/adaptive capacity), and along with exposure consider this an effective characterization of vulnerability. The only component of biological adaptive capacity that is not included in this is evolutionary change, which operates over geologic time frames and is thus not as relevant in the management time frame for which we are examining vulnerability in this study.

Assessment of relative vulnerability, for example among managed wetlands (or other units) within a prescribed area, is a common approach to inform policy and management questions (e.g., USEPA 2011). It is usually unrealistic to define an absolute measure of vulnerability due to the complexities of ecological systems and lack of knowledge of relevant thresholds. However, relative vulnerability is well suited to identify, within an area or region of interest: (1) Which endpoints (e.g., loss of wetland area and loss of a specific ecosystem service) or assessment units (e.g., wetland types, watersheds, or ecoregion contexts) are estimated to be more (or less) vulnerable; and (2) why the resources are vulnerable, that is, which components appear to be contributing to the perceived vulnerabilities. Note that the ranking of relative vulnerabilities among the units within a system will pertain only to the analysis frame of that system. Relative results will change if applied to a different system or to the same area but with the inclusion of more (or fewer) assessment units. This highlights the importance of articulating the management questions being addressed, as this defines the analysis frame (i.e., the set of units being compared) as well as the answer to the question “the vulnerability of what to what” (Adger et al. 2004). There can be confidence in relative vulnerability answers if a consistent, structured, repeatable approach is applied, but the answers themselves—the values—are scalable.

A critical part of assessing vulnerabilities is understanding the spatial and temporal scales at which exposure and sensitivity/adaptive capacity may shift, potentially necessitating a different management response. Exposure can be expected to vary regionally as well as at the finer watershed scale due to both geographic differences in climate (Burkett and Kusler 2000, Garris et al. 2015) and in the topographic, geological, and other environmental characteristics that mediate how that climate translates into hydrologic patterns. Similarly, temporal variability occurs interannually, but also at the finer seasonal scale. For example, fluctuations between wet and dry years have been shown to impact wetland vegetation, resulting in reductions in emergent vegetation in wet years and increases in dry years (e.g., Winter and Rosenberry 1998). Sensitivity/adaptive capacity may also vary between wetland types. Wetlands that utilize substantial contributions from groundwater can be buffered against manifesting these community composition changes (Burkett et al. 2005). Climate change has also been found to drive seasonal changes in wetland hydrology and water stress that impact wetlands across regions (Dawson et al. 2003). The heterogeneity expected at the finer spatial and temporal scales of watershed or season has been revealed by high-resolution spatial and temporal models (Yu et al. 2015), confirming the need for careful attention to variability and how it is manifested across scales. Thus, for the development of a management-relevant wetland vulnerability framework, we examined the sources of variability in vulnerabilities to climate change across spatial (ecoregion, small watershed), temporal (annual, seasonal), and organizational (broad, national wetland types, regionally based HGM types, ecoregion-specific HGM types) scales. Only then can we integrate relevant climate change considerations into management and develop appropriate adaptation approaches.

This paper characterizes relative wetland vulnerabilities at these multiple spatial, temporal, and organizational scales, and presents a framework for assessing vulnerability organized along the dimensions of exposure and sensitivity/adaptive capacity (Schneider et al. 2001, Glick et al. 2011). The framework is further discussed as a tool to apply in the process of integrating climate change considerations into the planning, development, and prioritization of robust management strategies.

## Methods

### Selection and classification of wetlands and watersheds

We used the HGM classification system (Brinson 1993*b*) to construct a vulnerability framework because it classifies wetlands based on hydrology in combination with geomorphology (position in the landscape) and water source, and is therefore a good way to differentiate hydrologic patterns between wetland types (Shaffer et al. 1999) and wetland ecological functions (Brinson 1993*b*, Smith et al. 1995). Broad patterns of hydrology have been characterized in our Mid-Atlantic study area using this classification scheme (Cole et al. 1997, 2002, Cole and Brooks 2000, Hychka et al. 2013), which may represent differential hydrologic sensitivities to climate change (Winter 2000). However, because the National Wetland Inventory (NWI) is the most widely available representation of wetland resources across the United States, we utilized it as an inventory of wetland area and then bridged between these two classification systems by applying the cross-walk described in Wardrop et al. (2011). We grouped NWI-defined wetlands into three coarse HGM classifications: isolated depressions, slope wetlands, and riverine wetlands. Slope wetlands were further divided into headwater floodplains and riparian depressions, consistent with the HGM classification process prepared for the Mid-Atlantic (Brooks et al. 2011). This level of HGM classification generally differentiates primary source water contributions to the three main wetland classes as follows: precipitation (isolated depressions), groundwater (slope wetlands), and surface water (riverine wetlands). It also helps to categorize key ecosystem services. For example, riparian (riverine and slope) wetlands are better able to regulate stream-flow and provide water quality improvements, while isolated depressions are better known for their ability to store larger amounts of carbon (Gilliam 1994, Bernal and Mitsch 2012, Wolf et al. 2013). The resulting inventory is presented in Table 1.

Two limitations of the NWI as applied in the Mid-Atlantic study area should be noted. First, the NWI-based estimate of wetland acreage is probably an underestimate, potentially capturing as little as 54% of total wetland acreage. A previous study in the Ridge and Valley province of Pennsylvania found that the NWI approach of interpreting vegetation types from aerial photography underestimated the numerous small, forested wetlands present (Wardrop et al. 2007). In heavily forested watersheds or regions of the country, this potential underestimation would have to be taken into account when relative vulnerability results are integrated with wetland acreage profiles for interpretation of relative risks in support of decision-making or setting of management priorities. Second, the NWI utilizes the Cowardin et al. (1979) classification system, based largely on descriptions of wetland vegetation types, in addition to soils and hydrology. Thus, NWI classification groups are not necessarily linked to wetland functions, and if used, a cross-walk to a functionally based classification centered around key wetland hydrologic characteristics should be sought.

**Fig. 1.**
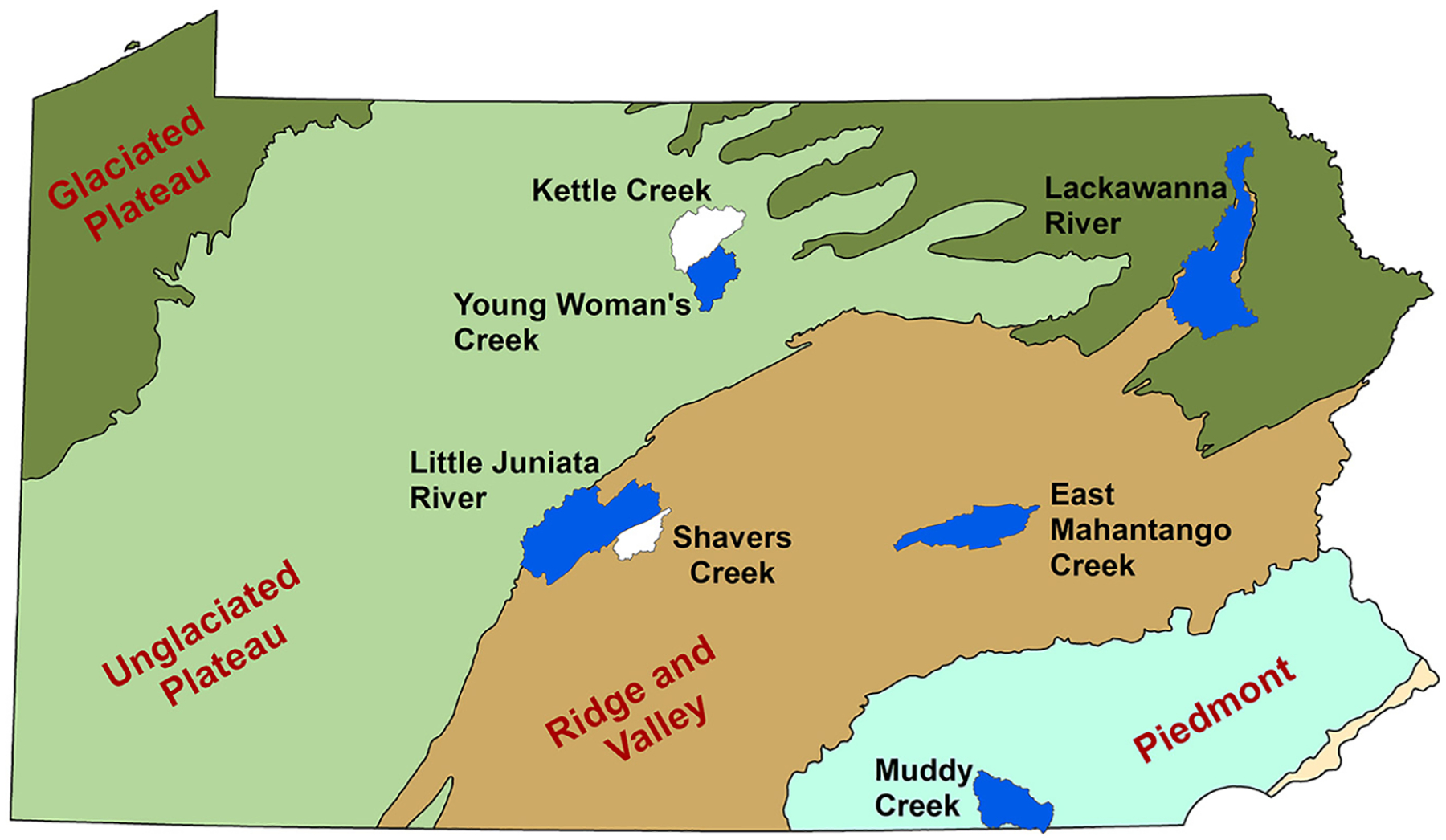
Locations of the seven watersheds used in the study and their respective ecoregions.

The study area included seven HUC-12 watersheds in the Susquehanna River basin of Pennsylvania, selected because of the extensive existing wetlands database available from the Penn State Riparia Research Center (Brooks et al. 2013), in combination with the availability of the Penn State Integrated Hydrologic Model (PIHM; Qu and Duffy 2007, Bhatt et al. 2008) for those watersheds. The seven study watersheds span four ecoregions and a range of geo-hydrologic conditions, land cover types, and patterns of anthropogenic activity (Fig. 1), although the distribution of watersheds among ecoregions was uneven.

The Susquehanna Basin (71,223 km^2^) includes four ecoregions: the Ridge and Valley (24,818 km^2^), the Glaciated Plateau (24,963 km^2^), the Unglaciated Plateau (14,806 km^2^), and the Piedmont (6636 km^2^) with a large portion of its stream kilometers in first- and second-order segments. It thus provides a full range of wetland types, capable of providing a wide range of ecosystem services such as nutrient cycling, sediment retention, water purification, flood control, and provision of critical habitat (Wardrop et al. 2011).

Shavers Creek, East Mahantango Creek, the Little Juniata River, and the lower portion of Lackawanna are located in the Ridge and Valley (R&V) physiographic province, characterized by heavily farmed, broad valleys and forested ridge tops. The R&V includes karst geology, where the weathering of soluble (e.g., carbonate) bedrock results in a complex and often poorly understood subsurface drainage. Karst features occur in the Shavers Creek and Little Juniata River watersheds, but not in the East Mahantango Creek watershed. Thus, there are watershed-scale geologic differences (e.g., watersheds with limestone vs. shale valley) and also land cover differences (watersheds with a high percentage of developed land cover) within this ecoregion that can be relevant to hydrology.

Kettle Creek and Young Woman’s Creek are located in the Unglaciated Allegheny Plateau (UG), characterized by steep hill slopes, narrow valleys, and relatively low amounts of farming and urban development. Muddy Creek is located in the Piedmont (P), which is often associated with rolling hills and dendritic drainage patterns. Finally, the Lackawanna River spans two physiographic provinces, with its downstream area in the R&V and its headwaters in the glaciated (G) portion of the Allegheny Plateau. The Glaciated Plateau is characterized by rounded hills and ridges, broad valleys, and high densities of small wetlands (Brooks et al. 2013).

To investigate whether results at the scale of individual wetlands can be generalized to the larger landscape (e.g., would the same sensitivities/adaptive capacities for a given wetland type be consistent across an ecoregion?), we compared patterns in the components of vulnerability across both watersheds and ecoregions. Classification by ecoregion focuses on largerscale landscape drivers (e.g., climate), while grouping by watershed focuses on somewhat smaller-scale patterns related to land uses, geologies, or ecological interactions.

### Construction of hydrologic change scenario

**Fig. 2.**
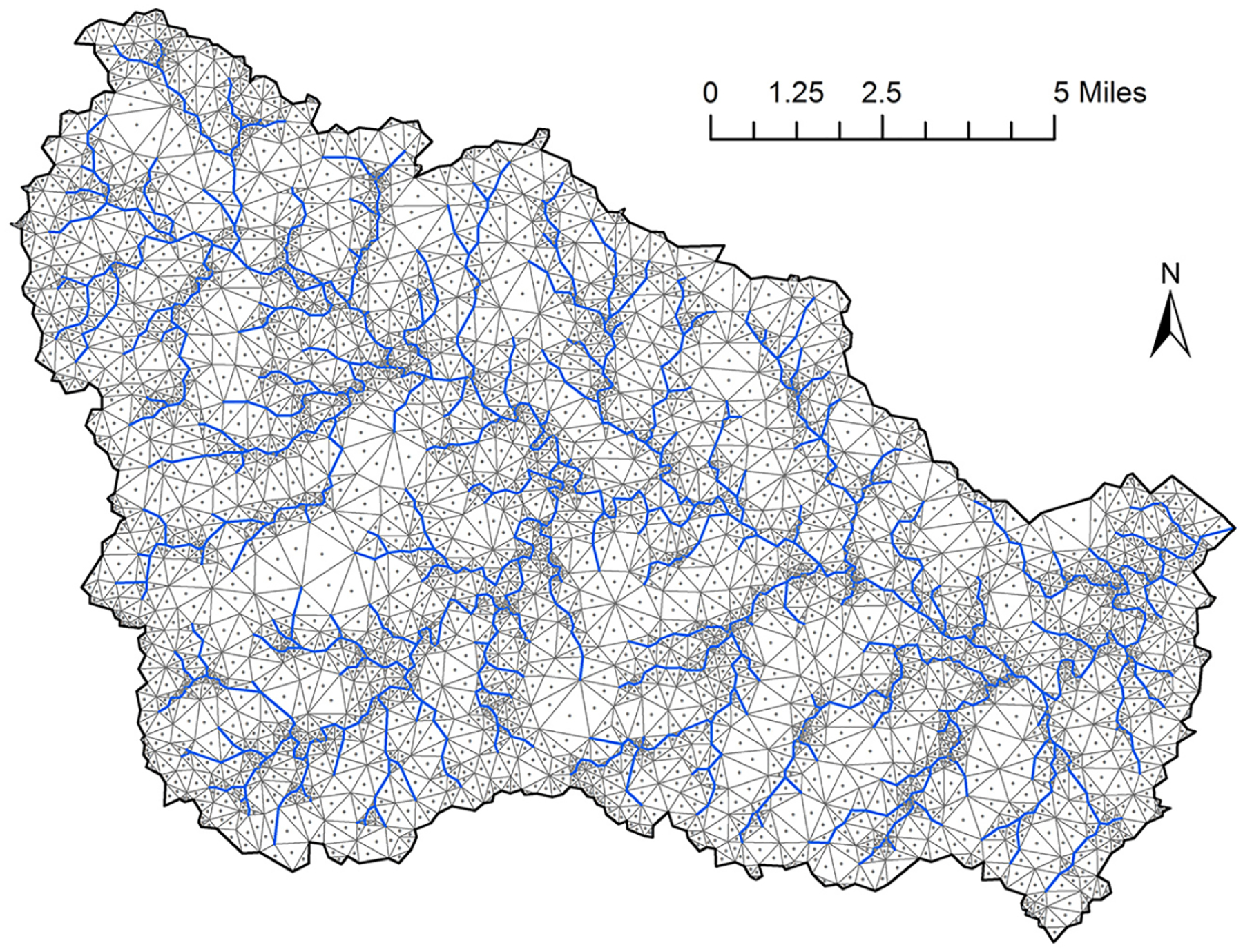
Penn State Integrated Hydrologic Model (PIHM) triangular irregular network (TIN) and associated centroids, shown for the Muddy Creek watershed.

The Penn State Integrated Hydrologic Model (PIHM; Qu and Duffy 2007, Bhatt et al. 2008) uses nationally available datasets, including USDA SSURGO soils data, land cover, USGS stream network and elevation data, and forcing variables from NASA Land Data Assimilation Systems (NLDAS) to construct watershed models, which we used to simulate historic (1979–1998) and project future (2046–2065) hydrologic regimes (www.pihm.psu.edu). Penn State Integrated Hydrologic Model future projections for this study were based on the SRES A2 emissions experiment using the MRI-CGCM2.3.2 climate model calibrated with observational data from weather stations in the Susquehanna River Basin (Yu et al. 2015). Each watershed model is composed of smaller modeling units referred to as triangular irregular networks (TINs; Fig. 2). TIN size varies across each watershed with hydrologic complexity, because they are drawn by the model so as to minimize variation in input variables within each TIN. TINs therefore tend to be smaller near streams and larger in the uplands and ranged from 7.3 hectares (18 acres) in the Young Woman’s Creek watershed to 40.5 hectares (100 acres) in the Little Juniata River watershed. All PIHM output data, including groundwater elevation data, are estimated at the centroid of each TIN for the 20-yr historic and future periods, and all hydrologic metrics are based upon this dataset. Finer resolution groundwater data were obtained using interpolation calculations in ESRI ArcGIS. Details regarding the PHIM modeling of our study watersheds can be found in Yu et al. (2015).

The model output used in this study is a measure of daily groundwater elevation above a constraining layer of bedrock. Uncertainties of model input data related to dataset development, data resolution, and/or simplifying assumptions will impact the accuracy of model outputs of groundwater levels and eventually the hydrology metrics that are calculated from that output. To minimize these, our study focused on the difference in groundwater levels between the historical and future scenarios, rather than on absolute groundwater level estimates.

To aid in interpretation of results, changes in groundwater regime in the future compared to historic scenarios were categorized as drier (future groundwater elevations more than 3 cm lower than historic conditions), wetter (future groundwater elevations more than 3 cm higher), or stable (future groundwater within ±3 cm of historic conditions). This value of ±3 cm represents 10% of the 30 cm rooting zone commonly used as a saturation depth in wetland characterization and was selected as a plausibly significant change even allowing for some natural variability that occurs in otherwise stable wetland areas. An alternative value to characterize this “stable” range of variation could easily be selected if, for example, more detailed or site-specific information justified it. Categorization of wetland groundwater level changes was done for annual and seasonal time periods, with seasons defined as follows: winter (December, January, February), spring (March, April, May), summer (June, July, August), and fall (September, October, November).

### Assessment of vulnerability

In the case study presented here, exposures (E) were hydrologic variables that integrated the primary climate change influences of temperature and precipitation, and were ecologically relevant to the selected wetland attributes (described in the next paragraph). The sensitivity/adaptive capacity (S/AC) metrics measured changes in the selected wetland attribute as a result of changes in the exposure metric. E and S/AC metrics for each attribute were normalized, and then depicted on a two-way graph, allowing us to identify vulnerability classes as low, moderate, or high, depending upon the quadrant within the exposure vs. sensitivity/adaptive capacity graph. That is, low E with low S/AC (lower left quadrant) yields low vulnerability, etc. These categorical relative vulnerabilities were identified and compared between different wetland units. Relative vulnerability was assessed by HGM type (summed across all watersheds studied), ecoregion (summed across all wetland types within each ecoregion), HGM type within ecoregion, and HGM type within watershed. The metrics selected for each attribute and the associated processes needed to calculate the metrics are described below.

We selected two wetland attributes on which to focus: wetland extent and plant community composition. Because in this case study we were not working directly with wetland managers in our study area, we chose typically meaningful attributes. However, for application of this approach in an actual management context, selection of wetland attributes may need to be reviewed based on specific management goals and objectives.

Wetland extent (e.g., total area that satisfies the ecological criteria of a wetland ecosystem) was chosen because it is common among all wetland types, is visually obvious and measurable, should respond to changes in hydrologic regime, and would represent a loss or gain of ecosystem services/functions via a change in area capable of providing such services/functions. Plant community composition was chosen because it is a driver for other functional processes (e.g., carbon cycling) and may be an earlier “signal” of impacts on wetland condition because changes in plant community composition might precede changes in extent. It has the added advantage of being commonly used as a condition assessment indicator (Fennessy et al. 2007).

**Fig. 3.**
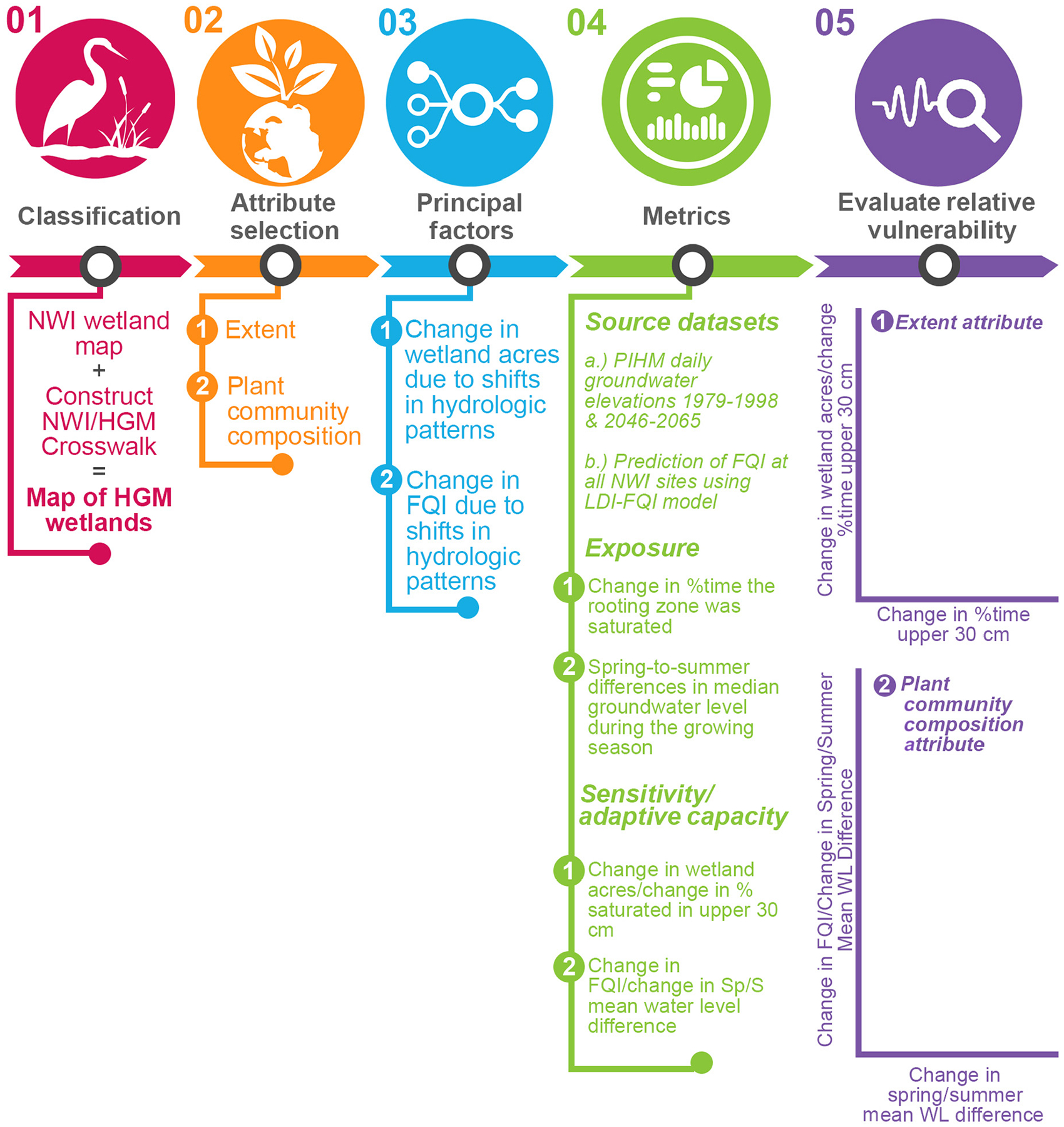
Methodology for the characterization of relative vulnerability for each of two attributes: wetland extent and plant community composition.

In general, once the attributes of interest are identified, methodologies for the characterization of exposure and sensitivity/adaptive capacity must be relevant to each; these are generally depicted in Fig. 3 and described separately as follows.

#### Extent.—

For extent, the selected measure of E was change in percent time the rooting zone was saturated (groundwater within the upper 30 cm, National Research Council 1995) during the growing season (spring–summer) between historic and future climate scenarios. We considered this metric ecologically relevant to the maintenance of wetland extent, because percent time of rooting zone saturation is commonly used to describe the hydrologic conditions in wetlands (Cole et al. 1997, 2002, Cole and Brooks 2000). Because the PIHM model did not reliably estimate absolute groundwater depth in the modeled watersheds, an absolute depth of 30 cm could not be used to represent the rooting zone. To compensate, we constructed a watershed-specific ecological calibration protocol by first determining the depth at which at least 75% of the modeled NWI wetland acreage was saturated at least 30% of the growing season in the historic climate scenario. This rooting zone depth was then used as a basis for calculating percent time the rooting zone was saturated under future conditions.

The S/AC metric for response of wetland extent is the rate of change in wetland acres. This is calculated as per unit change in percent time saturated during the historic climate scenario.

#### Community composition.—

For community composition, the ecologically relevant hydrologic metric (E) was the spring-to-summer differences in median groundwater (GW) level during the growing season. Spring mean water levels can be expected to play a major role in the establishment of various species, while summer mean water levels influence the energy available for asexual and sexual reproduction. The difference between these two levels represents the variability in conditions between these critical growing seasons and can be interpreted as posing a potential challenge to a number of native and specialist species. We calculated intra-annual differences between spring median water level and summer median water level for each HGM type within each watershed, under both historic and future conditions (Table 2). A positive value indicates that conditions are drier in summer than spring, and negative values represent conditions that are wetter in summer than in spring. E, with respect to climate change, were estimated as the average magnitude of change in this metric between historic and future conditions, expressed as a percent change relative to historic conditions (Table 2).

The S/AC metric for community composition response was estimated as the change in the Floristic Quality Index (FQI) relative to changes in the spring–summer GW levels. The FQI, originally developed by Swink and Wilhelm (1979, 1994), uses measures of ecological conservatism and richness of the native plant community to derive an estimate of habitat quality. It has been extensively tested across a large number of wetland types and regions (Bourdaghs et al. 2006, Jog et al. 2006, Miller et al. 2006, Nichols et al. 2006, Cretini et al. 2012) and is widely viewed as a reliable, stable, and informative expression of plant community composition and structure.

However, large datasets with synoptic measures of both hydrology and FQI are not widely available, nor were FQI data available for many of the wetlands in our modeled watersheds. Fortunately, land use data are available for all of the modeled watersheds, and previous studies have demonstrated significant responses in FQI with anthropogenic changes in land cover (Cohen et al. 2004, Mack 2006, Miller et al. 2006). Such anthropogenic disturbances in the landscape have been shown to manifest in hydrologic patterns, such as increased flashiness, more pronounced wetter or drier conditions, and disconnection between floodplain and stream hydrology (Mensing et al. 1998), which are similar to the types of hydrologic changes expected with climate change. Measures of anthropogenic disturbance could thus be used to predict measures of plant community composition that are relevant to climate change, since the primary (hydrological) mode of action is analogous.

We used the Landscape Development index (LDI) as an expression of anthropogenic disturbance that has been shown to be highly correlated with FQI (Mack 2006, 2007). We calculated LDI by multiplying land use percentages with a weighting factor related to the ecological energy needed to maintain that use (Odum and Odum 1975, Brown and Vivas 2005) for a 200 m radius surrounding each wetland site (Gunsch 2008).

Expecting that the relationship between LDI and FQI may be specific to an HGM class or ecoregion, or both, we constructed individual linear regressions for LDI vs. FQI by HGM type and ecoregion by using a dataset for which both were available for a relatively large number of sites. We used a dataset of 200+ wetlands in the Mid-Atlantic Region from Penn State’s Riparia Research Center (Brooks et al. 2013) that covered the four ecoregions and three HGM types represented in this study. Because floristic quality can be negatively affected by impacts such as buffer width, direct discharges, and other stressors not explicitly accounted for in the LDI, we used only points that demonstrated floristic quality minimally diminished by the presence of additional stressors outside of the LDI. To identify these otherwise “minimally impacted” sites (sensu Stoddard et al. 2006), we split sites into groups based on 10 units of LDI (for example, there might have been six sites with an LDI between 200 and 210) and only used the sites with the two highest adjusted FQI scores from each group. After cleaning the dataset in this manner, we regressed FQI vs. LDI. Regressions were done for all sites in Penn State’s Riparia reference collection and stratified by HGM type, ecoregion, and ecoregion/HGM type; the results are presented in Table 3. The high number of slope wetlands in the Ridge and Valley dominate the dataset, resulting in only three regression models with significance levels of *P* < 0.001: the models for “ceiling values” across all sites (*n* = 92), across all slope wetlands (*n* = 77), and across all Ridge & Valley sites (*n* = 72). In spite of non-significant *P* values for the remaining models, we retained them for use in the analysis, since they represent a relatively extensive dataset and exhibit *R*^2^ and *P* values that are consistent with other studies relating land cover to floristic measures. Two ecoregion–HGM type combinations (2 of 12) have no data to estimate a valid regression relationship; thus, for isolated depressions in the Piedmont and the Unglaciated Plateau, we used the model for isolated depressions in the Ridge and Valley. Finally, to produce our working dataset, we used these models to predict FQI at each of the NWI wetlands across all of the study watersheds.

As discussed above, the S/AC metric for community composition response was estimated as the change in the FQI relative to changes in the spring–summer GW levels. We retained the assumption that plant communities, and their hydrologic associations, would vary primarily by HGM type. Once we had populated all of the NWI wetlands across all of the study watersheds with FQI values, we then individually matched them with the site’s spring-to-summer differences in median groundwater levels, as calculated from the PHIM results for the 1979–1998 time period. We were interested in the distribution of FQI values with respect to this hydrologic metric as a measure of “sensitivity”, that is, what was the fidelity of a high-quality plant community (as indicated by a relatively high FQI) to a range of hydrologic conditions. This required four steps: (1) We selected sites with the top 10% of FQI values in any one HGM type as indicative of high-quality plant communities; (2) we plotted the distribution of high-quality plant communities across the entire range of spring-to-summer differences in median groundwater levels for that HGM type; (3) we assessed distribution measures of median, skewness, and kurtosis as descriptive of the fidelity of high-quality plant communities to hydrologic conditions in each HGM type; and (4) we individually normalized each distribution measure and combined the results into a final indicator of sensitivity. For example, the most sensitive plant community was that of slope-headwater floodplains (sensitivity of 3). For this HGM class, the distribution of high-quality plant communities across the entire range of spring-to-summer differences in median groundwater levels exhibited: (1) a low median, meaning that the high-quality plant communities occurred at very low spring-summer differences in median groundwater levels; (2) the distribution was highly skewed to the right, meaning that most of the high-quality plant communities occurred in wetter conditions; and (3) kurtosis was the least platykurtic, meaning that the distribution had a higher and sharper peak than the other HGM classes, indicating a high fidelity of the high-quality plant communities with a specific hydrologic condition. The table of median, skewness, and kurtosis values, and their interpretation, can be found in Appendix S1: Table S4.

### Inventory of relative vulnerabilities (vulnerability profiles)

To put the estimates of relative wetland vulnerabilities into a wetland management-relevant context, we produced “vulnerability profiles,” representing an inventory of relative wetland vulnerabilities for each study watershed for the community composition attribute. We used the results associated with the attribute of community composition as an exemplar, in part because it showed a greater range of response than did the attribute of extent in our study. However, the same process could (and would) be applied to any attributes used to assess vulnerability. We used the quantitative distribution of wetland acreage by HGM type (i.e., landscape (or wetland) profiles as per Bedford 1996, Gwin et al. 1999) and then applied the vulnerability class (low, high, or moderate) calculated for each watershed/HGM type.

### Relative magnitude of climate change hazard

As a yardstick for evaluating the magnitude of the management responses that might be called for relative to wetland climate change impacts, we compared responses in the E metric used for the community composition attribute, that is, the change (historic to future) in spring–summer mean groundwater level differences driven by climate change, to previous work that showed a similar response of increased spring–summer median water level differences associated with anthropogenic disturbance (Hychka et al. 2013). This was done as a graphic comparison for each of the three HGM wetland types.

## Results

### Distribution of wetlands within watersheds

Total wetland area and total number of wetlands varied across the seven study watersheds (Table 1). The average NWI wetland size in the study watersheds was 1.2 ha, with riverine wetlands averaging 2.6 ha, slope wetlands averaging 1.2 ha, and isolated depressions averaging 0.3 ha. Of the approximately 1200 individual NWI wetlands included in the seven study watersheds, 14% were classified as riverine wetlands, 63% as slope wetlands, and 23% as isolated depressions. On an area basis, of the approximately 1416 hectares (3500 acres) of NWI wetlands, 30% were classified as riverine wetlands, 63% as slope wetlands, and 6% as isolated depressions (Table 1).

### Scenarios of hydrologic change

**Fig. 4.**
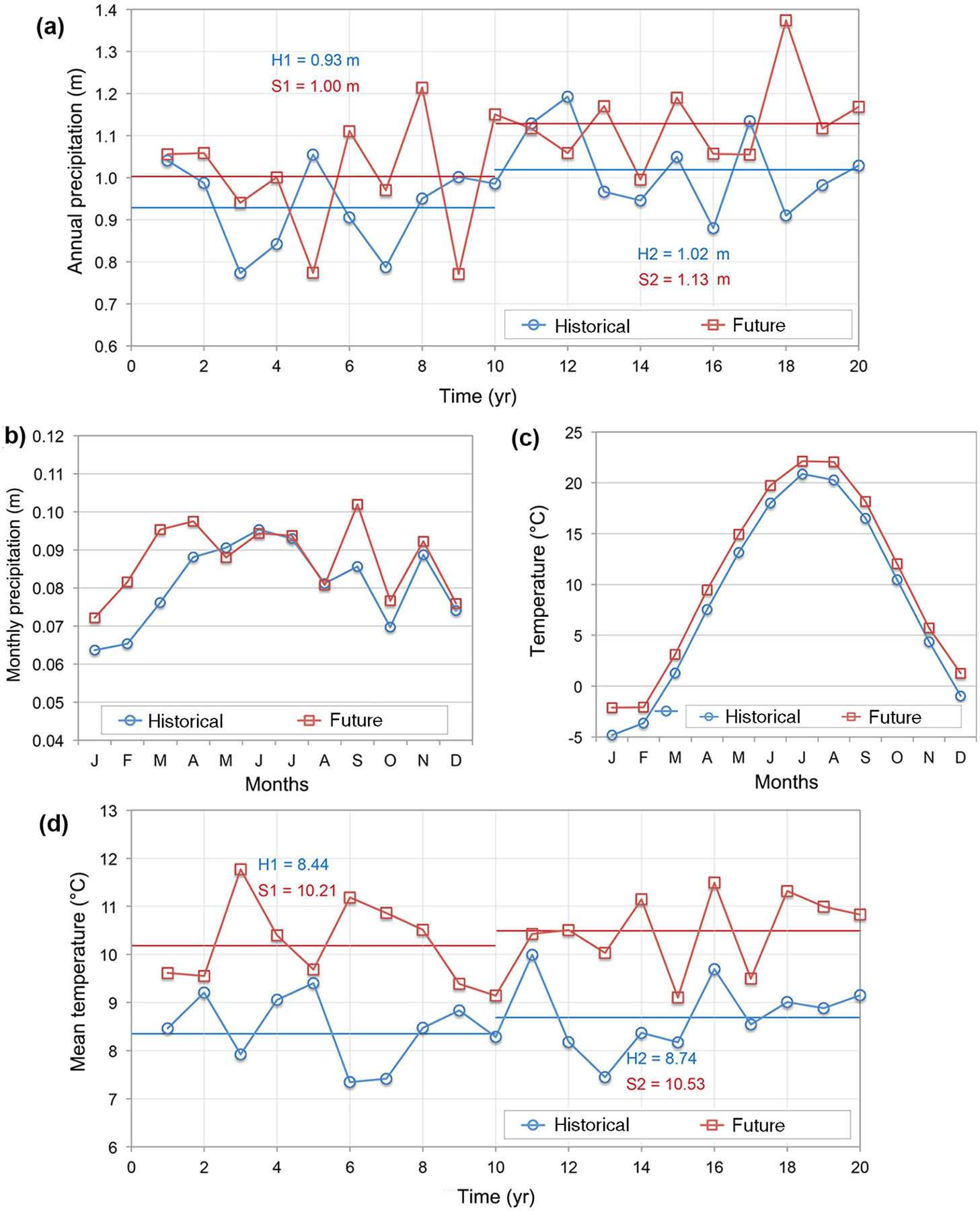
Modeled annual (a) and monthly (b) average precipitation and monthly (c) and annual (d) average temperatures over the 20-yr historical (1979–1998) and future (2046–2065) time periods, generated under the SRES A2 emissions experiment using the MRI-CGCM2.3.2 climate model.

Fig. 4 shows PIHM outputs of modeled historic and future temperature and precipitation, both annual averages (left panels) and seasonal patterns (right panels). Under the modeled scenario, future temperatures are projected to increase in all years and seasons (by an average of 1.8°C), though inter-annual variability is apparent. Model projections for precipitation are more variable among years although seasonally, average projections are for increases in fall, winter, and spring (10–11 mm) but with no change or slight decreases in precipitation during summer months (−1.5 to 3.5 mm).

Projected changes between simulated historical and future groundwater depth (assuming a future scenario of warmer, wetter conditions) indicated approximately half (51%) of the study area (seven study watersheds totaling 2466 km^2^) would see average annual groundwater changes of 3 cm or less (stable) (Table 4). Thirty-seven percent of the study area averaged greater than a 3 cm increase (wetter) in groundwater levels on an annual basis, and only 11% of the study area averaged greater than a 3 cm decrease (drier). A seasonal analysis of the wetter/stable/drier trends for the modeled watersheds indicates that while more than half the study area was wetter during the winter, spring, and fall, 70% (1726 km^2^) was drier during the summer. These results suggest future groundwater levels will experience a magnification of seasonal extremes, with historically wet periods getting wetter and historically dry periods getting drier. It should be noted that most assessments of climate change vulnerability are based on annual changes in groundwater levels; our assessment highlights the importance of investigation at a finer temporal grain (i.e., seasonally).

An analysis of annual groundwater changes considering only NWI wetland areas indicates similar overall trends of wetter/stable/drier as those observed for the entire watersheds (Table 5). During the summer, less wetland area appears to be getting drier than in the surrounding watershed. Broad HGM grouping of wetlands indicates little differentiation of wetter/stable/drier trends. Riverine wetlands, however, indicate a higher level of hydrologic stability annually, but become drier during the summer compared to isolated depressions and slope wetlands.

### Assessment of vulnerability

#### Extent.—

**Fig. 5.**
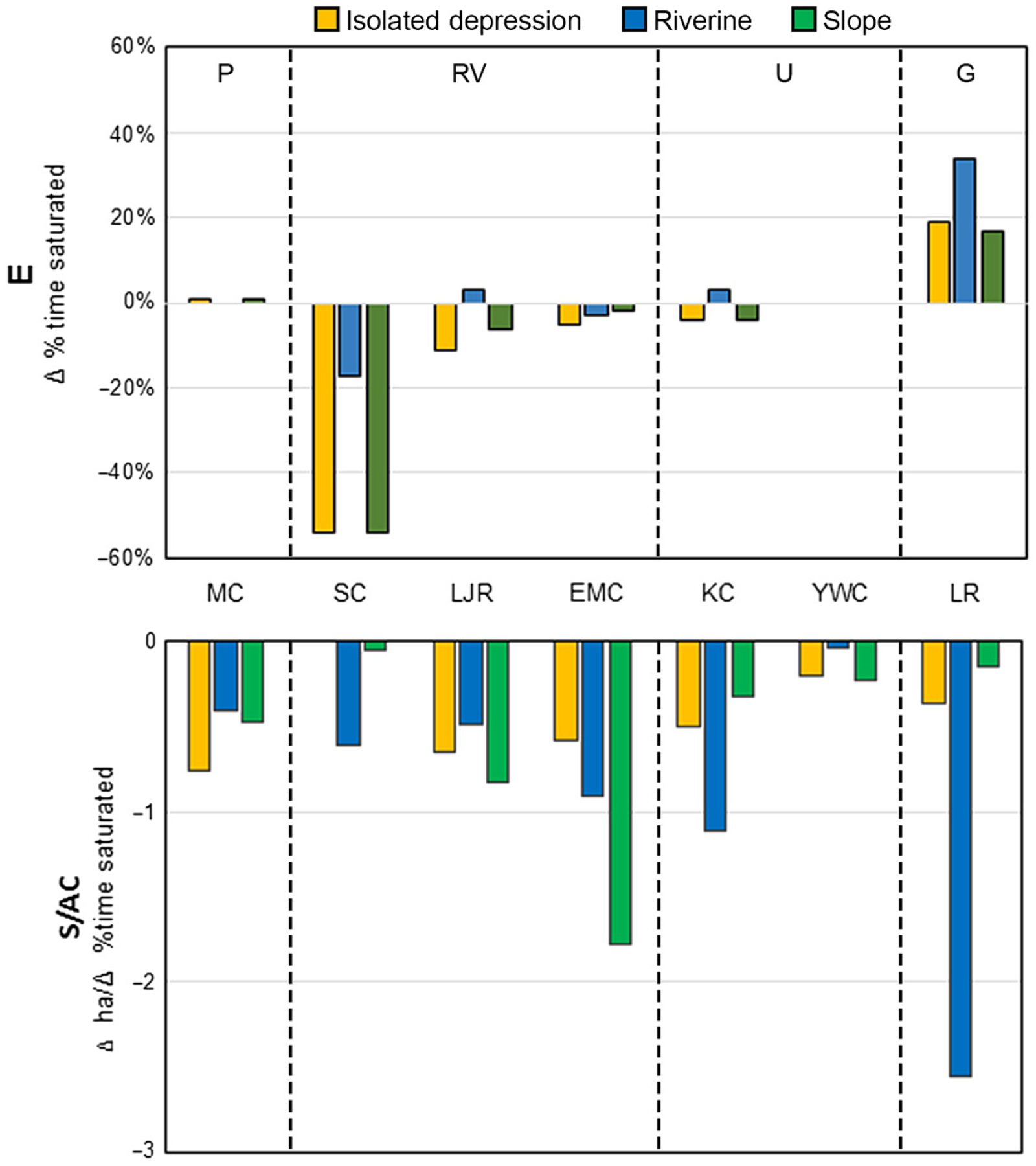
E (top) and S/AC (bottom) metrics for the attribute of wetland extent, by HGM type within each watershed. MC, Muddy Creek; SC, Shaver’s Creek; LJR, Little Juniata River; EMC, East Mahantango River; KC, Kettle Creek; YWC, Young Woman’s Creek; and LR, Lackawanna River watersheds.

The E metric (change between the historic and future climate scenarios in percent time the rooting zone was saturated during the growing season) exhibited high variability among HGM types and individual watersheds (Fig. 5, top), ranging from a strong positive change (wetter in the future) for the Lackawanna River to a strong negative change (drier in the future) for Shavers Creek. Within-ecoregion differences were apparent for the Ridge and Valley watersheds, but this is the only ecoregion for which a reasonable number of watersheds (>2 watersheds per ecoregion) were modeled. Shaver’s Creek showed the greatest decreases in percent time saturated in the rooting zone (−54% for isolated depressions and slopes to −17% for riverine wetlands), while the Little Juniata River and East Mahantango Creek indicated smaller decreases (−11% to −3%, depending on watershed and wetland type) to a slight increase (3%) for riverine wetlands in Little Juniata River. While there was variation across watersheds and ecoregions, when averaged by HGM type across the study area, increases in the percent time saturated in the rooting zone were similar for isolated depressions and slopes (6%), with a smaller increase observed in riverine wetlands (1%).

The S/AC metric for the extent attribute showed relatively small acreage changes across watersheds or ecoregions and HGM types (Fig. 5, bottom). The largest modeled change was a loss of approximately 2.4 riverine wetland hectares (6 acres) in the Lackawanna River watershed.

**Fig. 6.**
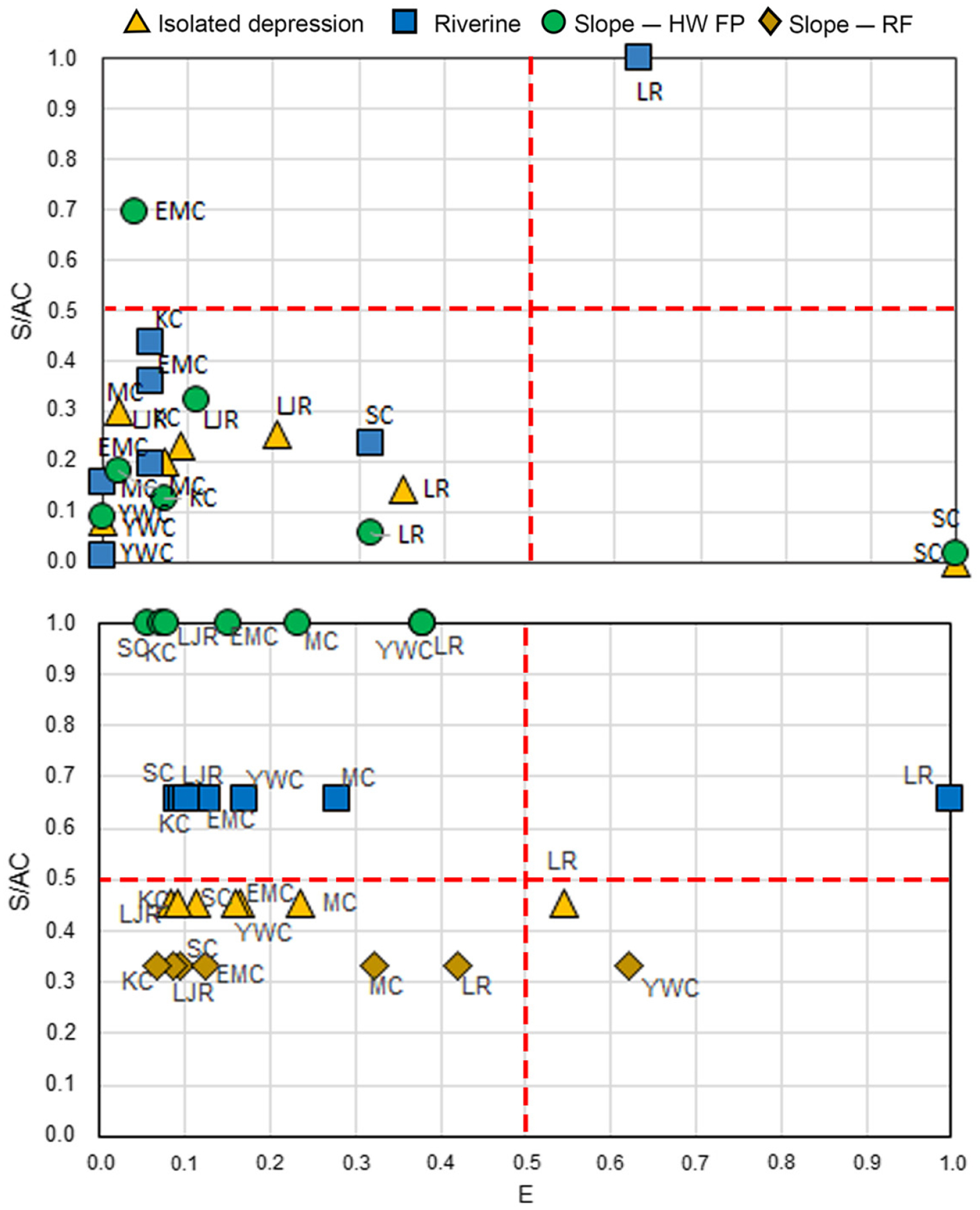
Relative vulnerability for the attributes of wetland extent (top) and plant community composition (bottom), by HGM type within each watershed. MC, Muddy Creek; SC, Shaver’s Creek; LJR, Little Juniata River; EMC, East Mahantango River; KC, Kettle Creek; YWC, Young Woman’s Creek; and LR, Lackawanna River watersheds.

Fig. 6 (top) displays the relative vulnerabilities based on the attribute of wetland extent, as a plot of E vs. S/AC to define a “vulnerability space.” These results are plotted at the finest scale that was evaluated, HGM type within each watershed. For extent, the presence of three relatively high values caused most of the wetland types in most of the watersheds to cluster in the lower left quadrant of the vulnerability space, indicating relatively low vulnerability and minimal differences between HGM types and watersheds. The lack of differentiation in the measure of change in extent among the majority of sites does not allow for a very useful ranking of relative vulnerability.

#### Community composition.—

**Fig. 7.**
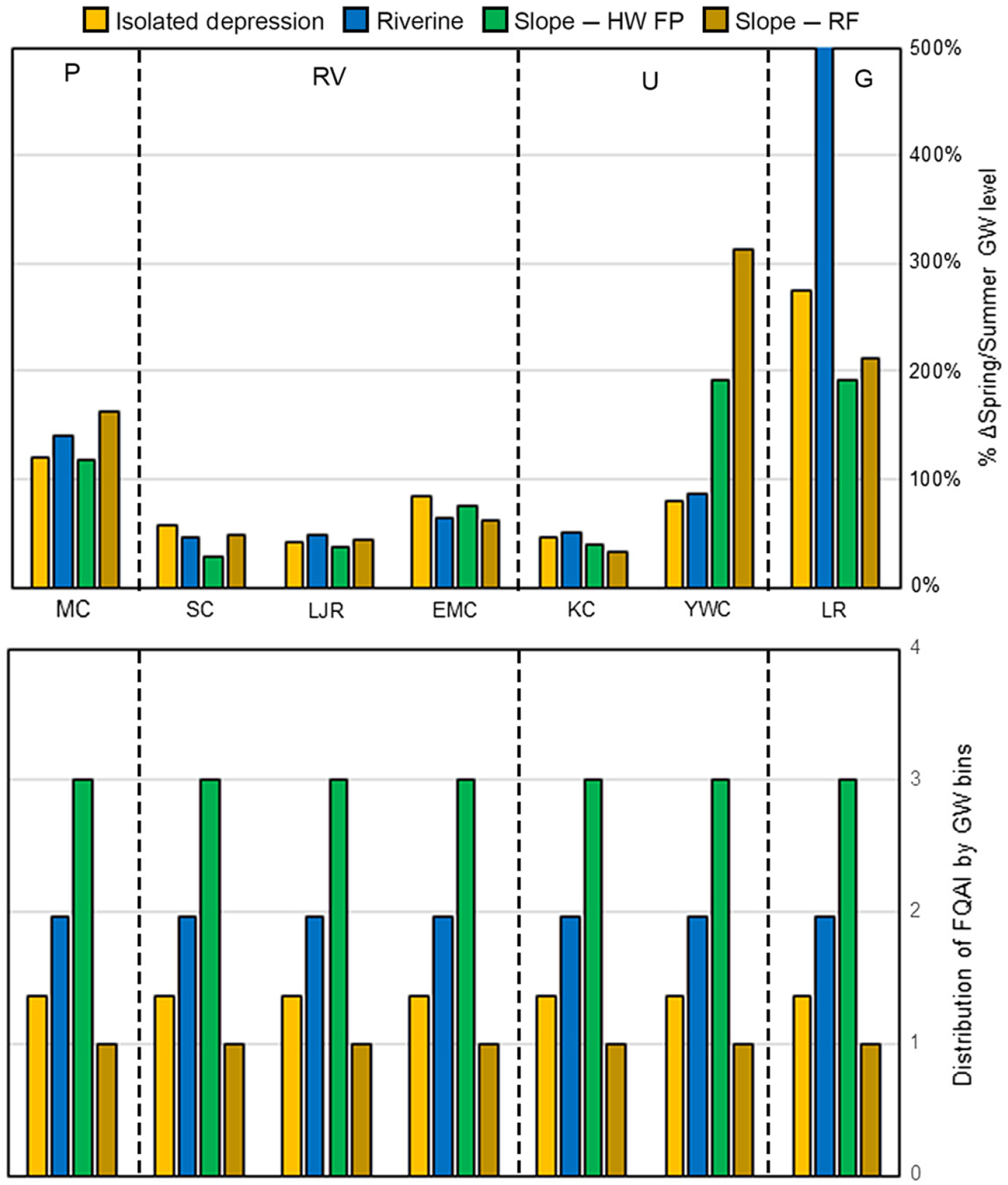
E (top) and S/AC (bottom) metrics for the attribute of plant community composition, by HGM type within each watershed. MC, Muddy Creek; SC, Shaver’s Creek; LJR, Little Juniata River; EMC, East Mahantango River; KC, Kettle Creek; YWC, Young Woman’s Creek; and LR, Lackawanna River watersheds. Notes: (1) Community composition sensitivity values for isolated depressions in the Piedmont and Unglaciated Plateau ecoregions were not available. Values for isolated depressions in the Ridge and Valley ecoregion were used as a proxy. (2) Community composition S/AC values were developed from reference datasets by ecoregion and are not watershed-specific. For ecoregions with multiple watersheds, only one set of values are provided. (3) Community composition S/AC for isolated depressions is equal to −0.646 and is scaled down to fit the above figure.

The spring-to-summer differences in median groundwater level showed high variability by watershed. Percentage changes in the E metric were greatest in Lackawanna River followed by Young Woman’s Creek and Muddy Creek, and lowest in the Ridge and Valley watersheds and Kettle Creek (Fig. 7, top). Throughout the evaluation of modeled groundwater data and metrics of vulnerability, the headwaters of the Lackawanna River in Northeastern Pennsylvania have consistently exhibited larger fluctuations in projected groundwater changes, sometimes an order of magnitude higher than the six other watersheds assessed in this study. The Lackawanna River watershed is the only location in our study that rests in the Glaciated Plateau ecoregion of Pennsylvania, meaning the underlying datasets (e.g. soils, bedrock, and land cover) used to populate the PIHM model are unique within our study sites. Additionally, because the climate forcing variables used in the model are downscaled datasets, expressing heterogeneity in both temperature and precipitation across the state, the Lackawanna River watershed received unique climate inputs because of its location northeast of all other study watersheds. These differences in model inputs, caused by the scale of the datasets used, highlight the importance of understanding the origins of modeled data when assessing vulnerability using the presented framework. Variability among HGM types within a watershed appeared low, except for Young Woman’s Creek, where E showed little change in the future for isolated depressions or riverine wetlands, but relatively large changes for the other types; and in Lackawanna, where the greatest changes were projected for riverine wetlands. Since the S/AC metric for community composition was calculated by HGM type, potential variation among watersheds or ecoregions could not be assessed (Fig. 7, bottom). Among HGM types, the response of slope-headwater floodplains was greatest and of slope-riparian depressions was the least.

Fig. 6 (bottom) shows the relative vulnerabilities based on the attribute of plant community composition, showing study wetlands by HGM type within each watershed. The metrics of E and S/AC in this case revealed a somewhat greater range of variation among wetland types in each watershed, leading to a more even distribution of watershed/HGM type units across the vulnerability space.

### Inventory of relative vulnerabilities

**Fig. 8.**
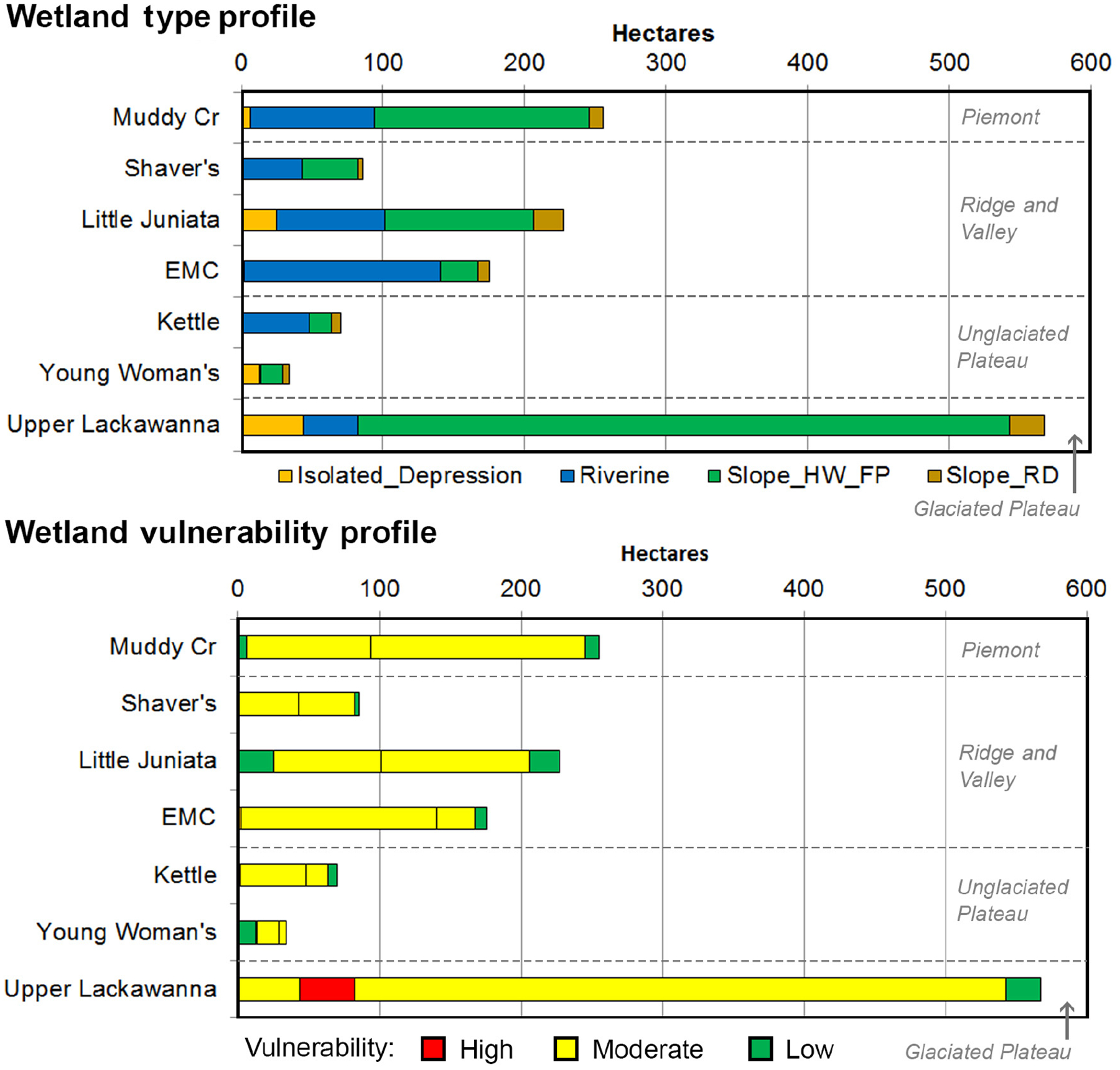
Relative wetland vulnerabilities, based on the attribute of plant community composition, displayed relative to the acreage of each wetland type in each watershed, according to the distribution of wetland acres in each watershed by HGM type (top panel), and as an expression of risk (bottom panel).

The inventory of relative vulnerabilities is presented as vulnerability profiles along with the corresponding HGM wetland profiles by watershed (Fig. 8). A complete tabulation of the vulnerability inventory (based on community composition) is also provided, consisting of wetland acreages and vulnerabilities, along with the component E and S/AC values; these are presented by HGM wetland type, ecoregion, ecoregion by HGM type, and watershed by HGM type (see Appendix S1: Table S1). For reference, the component E and S/AC values for the extent attribute are also presented in Appendix S1: Tables S2 and S3, respectively.

The inventory highlights differential patterns across a matrix of HGM wetland types, ecoregions, and watersheds. For example, slope-headwater floodplain wetlands are most abundant in the Upper Lackawanna watershed and are moderate in their relative vulnerability to climate change (Fig. 8), resulting in an inventory dominated by moderate climate change vulnerability. Given the widespread abundance of slope-headwater floodplain and riverine wetlands among the study watersheds, and the moderate vulnerability estimated for many of these, many of the watershed wetland vulnerability profiles are dominated by moderate vulnerability wetlands, although the combination of exposures and response inputs that contributed to the particular vulnerability rating differed among HGM-watershed combinations (Figs. 5 and 7). Among all of the HGM by watershed categories, only riverine wetlands in Lackawanna were classified as highly vulnerable. The wetland vulnerability profile by watershed and HGM type shows relative consistency in the distribution of wetland vulnerabilities among watersheds within a single ecoregion (Fig. 8).

### Framework for assessing relative vulnerabilities

**Fig. 9.**
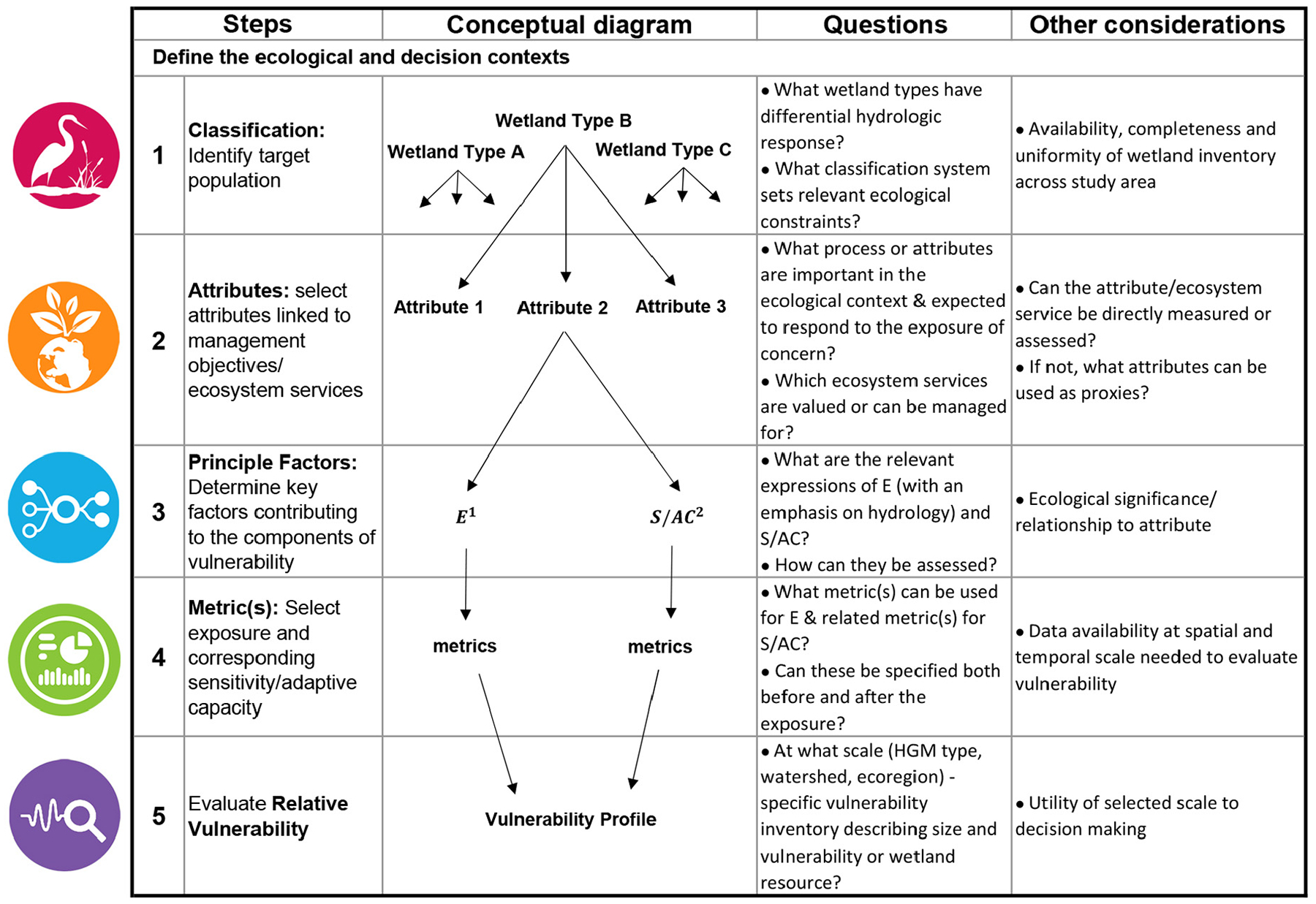
Relative wetland vulnerabilities framework.

Based on lessons learned from this exploratory case study, Fig. 9 presents our proposed framework for the assessment of relative vulnerabilities of wetlands to climate change. The framework is structured as a sequence of five steps that lead a manager through construction, implementation, and completion of the relative vulnerability assessment. A prerequisite for conducting the assessment is to articulate the ecological and decision contexts, which is commonly the first step of a management planning cycle (e.g., Kareiva et al. 2008, Glick et al. 2011). It includes understanding the goals and objectives of the management effort, describing the nature of decisions that the results of a vulnerability assessment must ultimately support. This becomes the basis for identifying attributes and endpoints of concern. For example, management targets might be a particular wetland type or an ecosystem service. If needed, this step can include the development of a simple ecosystem conceptual model (e.g., see USEPA 2012*a*, b) to identify ecological processes of priority concern in the wetland types of interest, in order to select the wetland attributes that will be used as the focus for assessing vulnerability. Although in this case study we did not work with a wetland manager to define a specific decision context, we made an overall assumption that our management goal was to preserve a broad spectrum of ecosystem services and that hydrology drives a variety of services. The five steps of the framework are further articulated below.

1. What wetland types have differential hydrologic responses? What classification system sets relevant ecological constraints?
  Step 1 of the framework identifies the target population and uses a relevant classification approach (in our case, HGM wetland classification) to express the ecological context around which the vulnerability assessment is organized, that is, the units for which relative vulnerabilities are being assessed. Hydrogeomorphic classification in particular focuses on the drivers of wetland structure and function as they are mediated through hydrology and landscape position, for example, the water sources, properties of geomorphic setting, and hydrodynamics (Brinson 1993*b*) that are likely to be effective organizers of ecological equivalence across wetland types. In practice, this classification step would include documenting the predominant HGM (or other) wetland types within a region, describing their functional attributes, and linking these with expected climate change sensitivities and corresponding exposures.
2. What processes or attributes are important in the ecological context, and expected to respond to the exposure of concern? Which ecosystem services are valued or can be managed for?
  Step 2 uses knowledge of the ecological and decision contexts plus the information from Step 1 to select one or more attribute(s) that will best reflect the processes of importance in the target wetland system, considering expected influences of climate change. In our case study, the attributes selected were extent and community composition, because they are both commonly measured in wetland systems, expected to be responsive to changes in hydrologic regime, and linked to the provision of numerous wetland ecosystem services.
3. What are the relevant expressions of exposure (with an emphasis on hydrology) and response (the combination of sensitivity and adaptive capacity)? How can they be assessed?
  Step 3 then “unpacks” the components of vulnerability—E and S/AC—in terms appropriate to the system and the management decision context (i.e., for the specific attributes that have been selected). For instance, as our example attribute is plant community composition, we expressed E as a hydrologic change from which we see a change in S/AC for plant community composition. Since hydrologic change integrates climate changes in temperature and precipitation, and is also the key driver of wetland structure and function, it may be that a single hydrologic factor can be used to describe the E component for assessment of relative wetland vulnerabilities to climate change (as was done in this case study). However, this may not always be the case, and then, Step 3 would be used to define a set of elements that together characterize the relevant climate change exposure (and similarly for sensitivity/adaptive capacity).
4. What metric(s) can be used for exposure (E) and response (S/AC)? Can these be specified both before and after the exposure?
  Step 4 follows with selection of an appropriate metric to measure changes in each E (hydrologic exposure) and S/AC (biological response) factor being considered. A large range of metrics may be relevant, and selection is likely to be influenced by data availability. It is essential to select the factor(s) and metric(s) only after picking the ecosystem attribute (Step 2), in order to ensure that the selection is ecologically meaningful in the context of that attribute.
5. What is the resulting vulnerability inventory? At what organizational scale (HGM type, watershed, ecoregion) should the inventory describing size and vulnerability of wetland resources be presented?
  Relative vulnerability (Step 5) is then calculated as a combination of the components of vulnerability described by the selected metrics. A simple linear combination (e.g., [E] × [S/AC]) can be used, yielding an estimate of relative vulnerability as a continuous variable (which was also done in this case study, see Appendix S1: Table S1). Alternatively, as in our example, a two-dimensional graph (one axis for each vulnerability component) can be used, and graphical interpretation can include setting breakpoints to define categorical descriptions (e.g., low/high or low/medium/high) for each component and for overall vulnerability.

### Comparison of climate change to historical anthropogenic disturbance

**Fig. 10.**
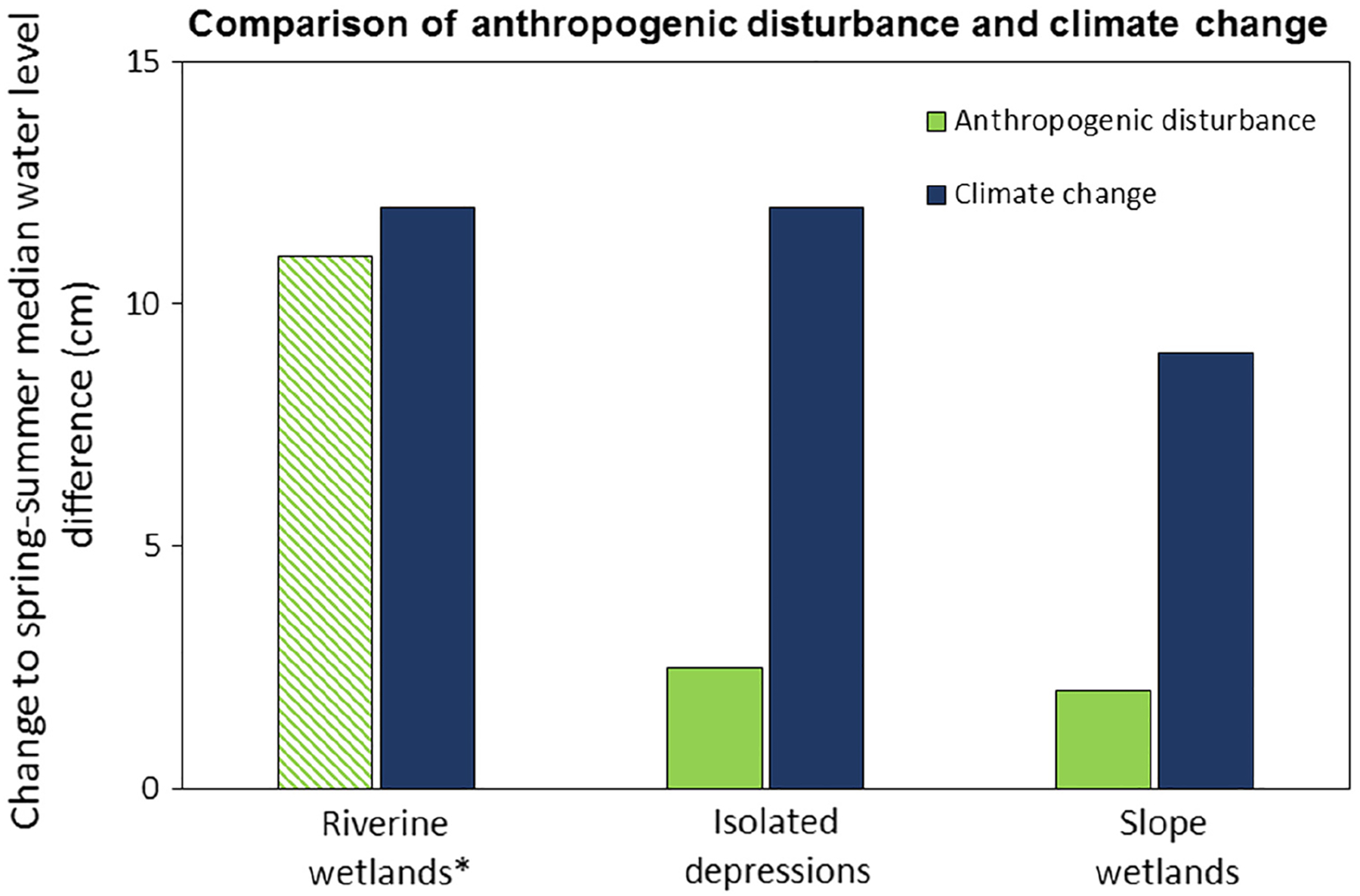
Comparison of differences in spring–summer median water levels resulting from anthropogenic disturbance and climate change. Values for anthropogenic disturbance represent the increase observed between pristine and disturbed wetland sites. Values for climate change represent the increase observed between historic and future climate scenarios. *The riverine value for anthropogenic disturbance is derived from single reference standard wetland and is plotted as an absolute value of change.

With respect to the relative magnitude of climate change compared to anthropogenic disturbance impacts on wetland hydrology, we found that the differences in spring–summer median water levels associated with climate change were slightly higher for riverine wetlands and four to five times larger for slope wetlands and isolated depressions than those resulting from anthropogenic disturbance (Fig. 10).

## Discussion

In this study, we implemented an in-depth modeling and analysis case study covering multiple wetland types in seven watersheds in Pennsylvania in order to develop and test an effective, consistent, and repeatable framework for assessing relative wetland vulnerabilities. Many previous wetland studies have postulated that the pathway for climate change vulnerabilities of wetlands is primarily through influences on the dynamic processes of wetland hydrology (Cox and Campbell 1997, Winter 2000, Poff et al. 2002, Dawson et al. 2003, Burkett et al. 2005, Erwin 2009, Pitchford et al. 2012, Garris et al. 2015). This study sought to investigate those assumptions and demonstrate characteristics of climate change-associated hydrologic changes as a basis for structuring a vulnerability framework that could be useful across diverse wetland types, regional contexts, and management situations.

Using a structured methodology to assess relative vulnerabilities of wetlands to climate change provides several benefits. (1) In a management context, the delineation of exposure vs. sensitivity/adaptive capacity may assist in illustrating what aspects of vulnerability can potentially be addressed through human-associated adaptation efforts, promoting a strategic approach to management. Information on which resources are vulnerable helps identify and prioritize among management targets, while information on why a particular resource is vulnerable informs on what could be done to reduce that risk (Glick et al. 2011, Stein et al. 2014). In addition, the resource limitations typical of most management programs necessitate some method of prioritization to help direct the deployment of the limited resources; delineating relative vulnerabilities is increasingly perceived as an effective and justifiable approach for accomplishing this. (2) Comparisons of relative vulnerabilities can be made across units of space, time, and ecological organization in an organized and systematic way. (3) This approach explicitly accounts for issues of scale, since the components of vulnerability are often assessed at different spatial and/or temporal scales. The question of scale is twofold: At what spatial, temporal, or organizational scale are relative differences in vulnerability observed? And can results at the finest scale be grouped into general statements that are appropriate at the regional or national scale?

We find that differences in exposure, measured as the hydrologic changes that manifest in response to changes in the primary climate variables of temperature and precipitation, occur at the finer spatial grain size of wetland type within a watershed, as well as among watersheds within an ecoregion. For example, with regard to the exposure metric used in association with the attribute of wetland extent, the four watersheds in the Ridge and Valley physiographic province demonstrate a higher intra-ecoregion variability than inter-ecoregion variability in general measures of watershed wetting/drying (Table 4). In contrast, the exposure metric used in association with the attribute of plant community composition was expressed a percent change relative to typical historic conditions. Thus, in this case, the variability in spring-to-summer groundwater level changes between watersheds within the Ridge and Valley ecoregion was reduced. This means that all the wetlands of one type, or all the wetlands in one region (e.g., Pennsylvania), are not necessarily similar in their relative vulnerabilities to climate change. Nevertheless, these detailed modeling results on expected hydrologic changes suggest that incorporation of vulnerability information into management decisions may need to occur at the watershed or wetland type level. This also illustrates the influence on vulnerability results of using hydrologic changes driven by climate change as the expression of wetland exposures, rather than the more readily available but larger-scale climate change factors of precipitation and temperature. Use of hydrologic exposure metrics more realistically reflects the scale at which the exposures impact the wetlands.

We have also demonstrated that annual changes in hydrologic parameters are too coarse to be ecologically relevant in the Mid-Atlantic. In many cases, annual averages show minimal future changes in hydrology due to climate change, which would underestimate probable wetland responses and mislead associated management decisions. The finer temporal grain of seasonal metrics is necessary to fully recognize potential climate change threats to the case study wetlands.

Exposure varies by HGM type, confirming the importance of choosing an appropriate ecological classification (in this case, one that is primarily based on differences in primary water source) as a basis for discerning differential responses. Measures of response for the attributes of wetland extent and plant community composition also vary between wetland HGM type and ecoregion (though we could not test watershed or ecoregional differences in the response metric for community composition). As a result, differences in relative wetland vulnerability persist at a very fine grain, even when a relatively spatially homogeneous forcing function of regionally downscaled projections of air temperature and precipitation is applied. Our case study illustrates the value of being able to integrate the broad effects of climate inputs into spatially explicit hydrologic patterns that are ecologically meaningful for wetlands, since it provides relative wetland vulnerabilities at a spatial scale approximating management decision-making (e.g., watershed scale). Our case study results confirm our initial premise that metrics of hydrology should be a defining element in the vulnerability assessment and that it is most appropriate to then structure a framework of assessment from that central principle.

The methodology we developed and tested in our case study of seven watersheds in the Mid-Atlantic successfully defined differences in relative wetland vulnerabilities to climate change-associated shifts in hydrology, indicating that it could be used to guide a manager to the identification of the most (and least) vulnerable wetlands in any given watershed. But given the diversity of the responses we found by wetland type and watershed, we could not generalize from the specific case study results to a set of prescribed relative vulnerabilities that could be applied regionally or nationally. Instead, we interpret our relative wetland vulnerabilities framework (Fig. 9) as a scaffold of systematic questions and considerations, applicable across wetland types and regions, that can guide a manager through the assessment of relative vulnerabilities of wetlands (differences between wetland types, ecoregions, watersheds, etc.), given the specification of a particular exposure, preferably one mediated through applicable components of wetland hydrology.

We recognize that intensive data, such as the modeled, fine-scale hydrologic scenarios we constructed, are not widely available and require resources for the development beyond the scope of many management programs. The goal of conducting a data-intensive study was primarily for the purpose of developing the framework. A secondary goal is to assure that the framework can be used at any scale of data intensity, so that it accommodates the need to make well-informed management decisions in a timely manner using the best available information, no matter the level of available resources. The steps and questions developed for the relative wetland vulnerabilities framework were crafted to fulfill this goal and be addressable at multiple levels of data richness. One can proceed through the framework in sequence, with inputs at each stage ranging from the narrative to the quantitative.

Using our case study as an example, at the step (1) of defining a classification of the study wetlands, a response could range from the conceptual (e.g., designating general categories of groundwater, surface water, and precipitation supported wetlands) to data-driven and data-specific (e.g., landscape profiles of acreage of HGM type by ecoregion). At either level, the answer can be consistent with the intent of the question at that step, in this case to define classes that are effective organizers of ecological equivalence across wetland types being assessed. Clearly, the final articulation of relative vulnerabilities will correspond with the level of detail of the component inputs, but by acting now with whatever level of information is available, using the framework can advance the goal of integrating climate change considerations into the management, protection, or restoration activities. Ongoing testing and application of the framework for other wetland types and under differing data availability and management scenarios (e.g., for coastal salt marshes, under data-rich and data-poor conditions, to inform restoration activities) are being undertaken to further advance this goal.

A word of caution is appropriate at this juncture. Relative vulnerabilities may vary by function or ecosystem service of interest. Impacts of anthropogenic disturbance across the recognized categories of wetland functions of habitat (e.g., Henning et al. 2007), water quality (Hemond and Benoit 1988, Ashby 2002), and biogeochemical cycling (e.g., Thiere et al. 2009) are expected to be variable, in some cases for any one wetland type, and may even be orthogonal to one another. For example, on the East Coast of the United States, more frequent and intense freshwater runoff events associated with climate change favor the spread of invasive *Phragmites* marsh grass. While *Phragmites* is better at filtration and sediment trapping than other grasses, it simultaneously reduces habitat quality for key bird species (USEPA 2012*b*). Therefore, assessment of more than one function may be recommended, in order to provide a more complete picture of relative vulnerabilities that enables more completely addressing management objectives.

The desire to develop a nationally applicable assessment of relative wetland vulnerabilities is largely driven by the value of being able to use results to inform multiple wetland programs operating at various spatial scales and from which results can be catalogued uniformly and compared. Our Mid-Atlantic study has shown in a rigorous way that case-specific relative vulnerability results are only meaningful with respect to a specified hazard and in the context of a particular space and timeframe. Thus, specific vulnerability results, even by HGM class, cannot simply be scaled up and applied to develop a national inventory. The very fact that projected climate changes driving wetland exposures vary greatly over regions within the United States (e.g., Smith 2004, IPCC 2013, Melillo et al. 2014, Blunden and Arndt 2015) speaks to the importance of applying the process within the context of regionally developed results. Instead, it is the critical elements of the structured framework that will put wetland relative vulnerability assessments on a comparable footing such that they can be scaled and applied regionally to develop a national inventory.

For projection of information supporting elevation of the inventory to larger regional or national scales, data would be needed to develop wetland profiles at those scales. The National Wetland Condition Assessment (NWCA) that is part of the U.S. EPA’s National Aquatic Resource Assessments (Scozzafava et al. 2011) has collected information on the distribution and abundance of wetland types based on a general HGM classification. The NWCA was conducted for the first time in 2011 and again in 2016, but at the time this paper was written, the updated 2016 results were not yet available. When results at this national scale become available, the potential utility of such national-scale wetland profile information could be tested for its value when aggregating regionally developed relative vulnerability results.

The presentation of relative wetland vulnerabilities as an inventory that relates vulnerabilities directly to the abundance of wetland acreage yields a risk management product that could be directly useful for informing management decisions. Understanding relative vulnerabilities provides key inputs to understanding the climate change problem (Adger et al. 2004, Fussel 2007, Berkhout et al. 2014) in a way that supports related adaptation planning (Dessai and van der Sluijs 2007, West et al. 2009, Julius et al. 2013, Stein et al. 2014). An inventory of relative wetland vulnerabilities categorized by HGM wetland type, and at a spatial scale (e.g., watershed, ecoregion, or other) that is relevant to management objectives, can be used to help decide where and how resources and efforts should be focused. In our Mid-Atlantic case study, the vulnerability profile by watershed and HGM type (Fig. 8) shows, for example, that in the Lackawanna River watershed where wetlands are very abundant, the dominant wetland type, slope (headwater floodplain), has a moderate vulnerability to spring–summer hydrologic changes resulting from climate change. A manager can now consider whether Lackawanna slope (headwater floodplain) wetlands should be protected because of their relative vulnerability, or whether management should focus on restoration efforts in other wetland types in the Lackawanna watershed that were found to be more vulnerable (e.g., riverine), in order to increase their resilience. Decisions such as where and how to expend resources, for example, whether to focus on the most vulnerable or least vulnerable resources, will be influenced by the particular management objectives and must consider other decision criteria such as social values, economic constraints, and legal considerations (e.g., Stein et al. 2014). It is thus key to recognize that the wetland vulnerability information developed in this framework can support setting priorities and making these and other management decisions, but does not dictate the decisions (Glick et al. 2011).

The potential for shifts in hydrologic patterns due to climate change is beyond what wetlands managers have experienced in the past (as illustrated in Fig. 10). This brings into question whether we have the necessary tools to construct effective management adaptations in the face of climate change impacts and highlights the importance of utilizing a uniformly applicable relative wetland vulnerabilities framework to focus planning, protection, and restoration efforts.

## Concluding Remarks

One of the goals of developing this relative wetland vulnerabilities framework and inventory is to help inform existing wetland management, regulatory, and monitoring programs to fulfill the national mandate to integrate climate change considerations and adaptation into their activities (Executive Order 13514). Future efforts in this project will extend our understanding of particular program needs and develop recommendations regarding how this framework and resulting vulnerability information can best be integrated into selected existing wetland programs, such as voluntary restoration programs at the state or regional level, as well as national management and regulatory programs such as the Clean Water Act Section 404 compensatory mitigation program, EPA Healthy Watersheds Program, and the National Wetland Condition Assessment. These programs differ in their mandates and goals, and in many of their key activities, so it is clear that the ways vulnerability information can be integrated into their activities are likely to differ. However, our study provides some insights that can guide a general approach, applicable across all programs. Despite the national scope of many such programs, on-the-ground implementation of program objectives and activities often occurs at the local or regional level. It is thus feasible and recommended that this relative wetland vulnerabilities framework be used to support programmatic adaptation going forward. This provides a key first step toward preparing for anticipated shifts in hydrologic patterns that are beyond current management experience, with the goal of preserving the valued ecosystem services provided by the nation’s wetland resources.

## Supplementary Material

Supplement1

Additional Supporting Information may be found online at: http://onlinelibrary.wiley.com/doi/10.1002/ecs2.2561/full

## Figures and Tables

**Table 1.** Study watershed areas grouped by ecoregion, with total number and area of NWI wetlands, and wetland distribution by HGM type (hectares).

Ecoregion	Watershed	Watershed area (× 10^2^ ha)	No. NWI wetlands	HGM wetlands (ha)				Total
				Isolated depression	Riverine	Slope-HW FP	Slope-RD	
Piedmont	Muddy Creek	360	191	6	88	151	10	255
Ridge and Valley	Shavers Creek	163	58	0.1	43	39	4	86
	Little Juniata River	843	531	25	77	105	21	227
	East Mahantango Creek	422	103	2	138	27	8	175
	Lackawanna River	808	877	200	92	1014	123	2576
Unglaciated Plateau	Kettle Creek	355	105	1.1	47	16	7	71
	Young Woman’s Creek	230	43	13	0.3	16	5	34
Glaciated	Lackawanna River	94	193	44	38	461	25	567
Study area		3275	2101	291	522	1829	202	3991

**Table 2.** Historic and future intra-annual difference between spring median water level and summer median water level (cm) by HGM type within individual watersheds.

Watershed (ecoregion)	HGM type	Average intra-annual difference†		Difference (future—historic)	Percent change (relative to historic)
		Historic (cm)	Future (cm)		
Muddy Creek (P)	Isolated Depression	3.3	7.3	4.0	119.4
	Riverine	6.5	15.6	9.1	141.1
	Slope-HW FP	6.6	14.5	7.8	118.1
	Slope-RD	3.1	8.3	5.1	163.0
Shaver’s Creek (R&V)	Isolated Depression	0.5	0.8	0.3	57.2
	Riverine	3.0	4.4	1.4	45.7
	Slope-HW FP	4.1	5.2	1.2	28.2
	Slope-RD	2.8	4.1	1.3	47.4
Little Juniata River (R&V)	Isolated Depression	33.3	47.5	14.2	42.6
	Riverine	25.9	38.6	12.6	48.6
	Slope-HW FP	28.5	39.1	10.6	37.1
	Slope-RD	30.6	44.0	13.4	43.9
East Mahantango Creek (R&V)	Isolated Depression	16.1	29.5	13.4	83.7
	Riverine	24.7	40.7	15.9	64.4
	Slope-HW FP	21.5	37.7	16.3	75.7
	Slope-RD	18.1	29.4	11.3	62.2
Kettle Creek (U)	Isolated Depression	41.7	60.8	19.1	45.7
	Riverine	30.5	46.2	15.7	51.5
	Slope-HW FP	39.9	55.4	15.5	39.0
	Slope-RD	30.1	40.2	10.0	33.3
Young Woman’s Creek (U)	Isolated Depression	15.7	28.4	12.7	80.6
	Riverine	0.6	1.1	0.5	85.7
	Slope-HW FP	5.8	16.9	11.1	191.3
	Slope-RD	0.6	2.3	1.8	313.5
Lackawanna River (G)	Isolated Depression	1.8	6.8	5.0	275.6
	Riverine	−0.7	2.9	3.6	505.7
	Slope-HW FP	2.6	7.5	4.9	191.4
	Slope-RD	1.8	5.5	3.7	213.0

*Note:* P, Piedmont; R&V, Ridge and Valley; U, Unglaciated Plateau; G, Glaciated Plateau.

† Average intra-annual difference between spring median water level and summer median water level. Positive value indicates water level is higher in spring than summer.

**Table 3.** Linear regression results used to construct LDI_200_-to-adjusted FQAI model (translator).

Ecoregion	HGM category	Original *n*	Ceiling *n*	Equation	*R* ^2^	*P*
	All Sites	268	92	*y* = −0.0392*x* + 54.106	0.27488	0.0001
	All Slopes	176	77	*y* = −0.0343*x* + 51.022	0.1822	0.0001
	All Isolated Depressions	27	14	*y* = −0.0433*x* + 53.809	0.32408	0.0336
	All Riverine	51	36	*y* = −0.0146*x* + 40.666	0.0817	0.0910
	Glaciated	39	27	*y* = −0.0194*x* + 50.549	0.04177	0.3147
	Unglaciated	46	28	*y* = −0.0103*x* + 40.307	0.01891	0.4753
	Ridge and Valley	162	75	*y* = −0.0377*x* + 50.921	0.30326	0.0001
	Piedmont	8	8	*y* = −0.0161*x* + 43.58	0.12513	0.4032
Glaciated	Slope	26	21	*y* = 0.0009*x* + 44.473	9.7E-05	0.9759
	Isolated Depression	4	4	*y* = −0.6459*x* + 130.26	0.35229	0.4065
	Riverine	9	7	*y* = −0.0343*x* + 50.171	0.33888	0.1734
Unglaciated	Slope	32	23	*y* = −0.0017*x* + 32.997	0.00021	0.9485
	Isolated Depression	0	0	NA	NA	NA
	Riverine	14	11	*y* = −0.0053*x* + 39.549	0.02705	0.6310
Ridge and Valley	Slope	114	63	*y* = −0.038*x* + 49.915	0.28158	0.0001
	Isolated Depression	23	11	*y* = −0.042*x* + 53.584	0.46747	0.0202
	Riverine	25	23	*y* = −0.0122*x* + 36.393	0.05078	0.2944
Piedmont	Slope	5	5	*y* = −0.0085*x* + 43.459	0.03067	0.7993
	Isolated Depression	0	0	NA	NA	NA
	Riverine	3	3	*y* = −0.0177*x* + 41.042	0.67339	0.3823

*Notes:* Models were as presented for all ecoregion × HGM categories, except for isolated depressions in the Unglaciated Plateau and Piedmont ecoregions. For these two categories, the model for isolated depressions in the Ridge and Valley ecoregion was used (as noted in *Methods/Assessment of Vulnerability/Community Composition*).

**Table 4.** Percent of study area classified as wetter (>3 cm increase), stable (3 cm or less change), or drier (>3 cm decrease) annually and during the summer.

Study watershed	Annual change (%)			Summer change (%)		
	Drier	Stable	Wetter	Drier	Stable	Wetter
Total study area (2466 km^2^)	11	51	37	70	19	11
Muddy Creek	0	51	49	100	0	0
Shavers Creek	48	46	6	57	38	5
East Mahantango Creek	9	65	26	74	16	10
Little Juniata River	20	45	35	50	32	18
Young Woman’s Creek	0	14	86	84	16	0
Kettle Creek	0	88	12	97	3	0
Lackawanna River—Glaciated	0	12	88	2	16	82

*Note:* Comparison based on average modeled groundwater level differences between historic and future climate scenarios.

**Table 5.** Percent of wetland area classified as wetter (>3 cm increase), stable (3 cm or less change), or drier (>3 cm decrease) annually and during the summer.

HGM category	Annual (%)			Summer (%)		
	Drier	Stable	Wetter	Drier	Stable	Wetter
Wetlands only within-study watersheds	4	50	45	51	19	30
Isolated depressions	7	48	45	38	24	38
Slope wetlands	3	47	50	39	22	39
Riverine wetlands	6	59	35	78	10	11

*Note:* Comparison based on average modeled groundwater level differences between historic and future climate scenarios.
