# Supplementary material for: Assessing the relative vulnerabilities of Mid-Atlantic freshwater wetlands to projected hydrologic changes
Source: Ecosphere. Author manuscript; Available in PMC 2025 Jun 30. (PMC12208506; doi:10.1002/ecs2.2561)
Supplement: Supplement1 [file NIHMS2089065-supplement-Supplement1.pdf]

Ecosphere
Assessing the relative vulnerabilities of mid-Atlantic freshwater wetlands to projected hydrologic changes
Denice H. Wardrop, Anna T. Hamilton, Michael Q. Nassry, Jordan M. West, and Aliana Britson

Appendix S1

Appendix Table S1. Inventory of Relative Wetland Vulnerabilities, Plant Community Composition Attribute.

	HGM Type	Wetland Hectares	E	S/AC	Relative Vulnerability	
					Value	Category
**Watershed**						
Muddy Creek (P)	Isolated Depression	15	119.4%	1.36	0.11	Moderate
	Riverine	218	141.1%	1.97	0.18	Low
	Slope-HW FP		118.1%	3.00	0.23	
	Slope-RS	398	163.0%	1.00	0.11	Low
Shaver's Creek (R&V)	Isolated Depression	0.3	57.2%	1.36	0.05	Moderate
	Riverine	106	45.7%	1.97	0.06	Moderate
	Slope-HW FP		28.2%	3.00	0.06	Low
	Slope-RD	107	47.4%	1.00	0.03	Moderate
Little Juniata River (R&V)	Isolated Depression	61	42.6%	1.36	0.04	Low
	Riverine	189	48.6%	1.97	0.06	Moderate
	Slope-HW FP		37.1%	3.00	0.07	Moderate
	Slope-RD	312	43.9%	1.00	0.03	Low
East Mahantango Creek (R&V)	Isolated Depression	4	83.7%	1.36	0.08	Low
	Riverine	342	64.4%	1.97	0.08	Moderate
	Slope-HW FP		75.7%	3.00	0.15	Moderate
	Slope-RD	87	62.2%	1.00	0.04	Low
Kettle Creek (U)	Isolated Depression	2.68056	45.7%	1.36	0.04	Low
	Riverine	116	51.5%	1.97	0.07	Moderate
	Slope-HW FP		39.0%	3.00	0.08	Moderate
	Slope-RD	55	33.3%	1.00	0.02	Low
Young Woman's Creek (U)	Isolated Depression	32	80.6%	1.36	0.07	Low
	Riverine	0.7	85.7%	1.97	0.11	Moderate
	Slope-HW FP		191.3%	3.00	0.38	Moderate
	Slope-RD	51	313.5%	1.00	0.21	Moderate
Lackawanna River (G)	Isolated Depression	109	275.6%	1.36	0.25	Moderate
	Riverine	93	505.7%	1.97	0.66	High
	Slope-HW FP		191.4%	3.00	0.38	Moderate

- Lacked sufficient data to estimate regression ($n \leq 4$); replaced with Ridge & Valley values in calculation of vulnerability.

Appendix Table S2. E metric results for the Extent Attribute: Change (historic to future with climate change) in frequency (as % time) of saturation in the rooting zone (upper 30 cm).

	Isolated Depression	Riverine	Slope
HGM	6%	1%	6%
Ecoregion			
Piedmont (P)	1%	0%	1%
Ridge & Valley (R&V)	-16%	-3%	-13%
Unglaciated Plateau (U)	-3%	2%	-3%
Glaciated Plateau (G)	19%	34%	17%
Watershed			
Muddy Creek (P)	1%	0%	1%
Shaver's Creek (R&V)	-54%	-17%	-54%
Little Juniata River (R&V)	-11%	3%	-6%
East Mahantango Creek (R&V)	-5%	-3%	-2%
Kettle Creek (U)	-4%	3%	-4%
Young Woman's Creek (U)	0%	0%	0%
Lackawanna River (G)	19%	34%	17%

Appendix Table S3. S/AC metric results for the Extent Attribute: Rate of change (acres/cm) in wetland acres per unit change in percent time saturated during the historic climate scenario.

	Isolated Depression	Riverine	Slope
HGM	-1.11	-2.14	-0.90
Ecoregion			
Piedmont (P)	-1.88	-1.00	-1.15
Ridge & Valley (R&V)	-1.26	-1.63	-2.54
Unglaciated Plateau (U)	-1.00	-1.89	-0.72
Glaciated Plateau (G)	-0.91	-6.31	-0.36
Watershed			
Muddy Creek (P)	-1.88	-1.00	-1.15
Shaver’s Creek (R&V)	0.00	-1.48	-0.11
Little Juniata River (R&V)	-1.59	-1.21	-2.03
East Mahantango Creek (R&V)	-1.44	-2.25	-4.39
Kettle Creek (U)	-1.24	-2.76	-0.80
Young Woman’s Creek (U)	-0.50	-0.08	-0.55
Lackawanna River (G)	-0.91	-6.31	-0.36

Appendix Table S4. Distribution measures utilized in the Sensitivity metric for Plant Community Composition. All measures are based on the distribution of high quality plant communities (defined as sites with the top 10% of FQI values in any one HGM type) across the entire range of spring-to-summer differences in median groundwater levels.

HGM Category	Median	Skewness	Kurtosis (Excess)
Isolated Depressions	10	0.30	-0.45
Riverine	11	0.53	-0.26
Slope –HW FP	7	0.77	-0.15
Slope - RD	12	0.13	-0.6
